# Flow cytometric analysis reveals culture condition dependent variations in phenotypic heterogeneity of *Limosilactobacillus reuteri*

**DOI:** 10.1038/s41598-021-02919-3

**Published:** 2021-12-07

**Authors:** Nikhil Seshagiri Rao, Ludwig Lundberg, Shuai Palmkron, Sebastian Håkansson, Björn Bergenståhl, Magnus Carlquist

**Affiliations:** 1 Division of Applied Microbiology, Department of Chemistry, Lund University, Lund, Sweden; 2 The Department of Molecular Sciences, Uppsala BioCenter, Swedish University of Agricultural Sciences, Uppsala, Sweden; 3 BioGaia AB, 103 64 Stockholm, Sweden; 4 Department of Food Technology, Engineering and Nutrition, Department of Chemistry, Lund University, Lund, Sweden; 5 BioGaia AB, 241 38 Eslöv, Sweden

**Keywords:** Industrial microbiology, Bacterial physiology, Applied microbiology

## Abstract

Optimisation of cultivation conditions in the industrial production of probiotics is crucial to reach a high-quality product with retained probiotic functionality. Flow cytometry-based descriptors of bacterial morphology may be used as markers to estimate physiological fitness during cultivation, and can be applied for online monitoring to avoid suboptimal growth. In the current study, the effects of temperature, initial pH and oxygen levels on cell growth and cell size distributions of *Limosilactobacillus reuteri* DSM 17938 were measured using multivariate flow cytometry. A pleomorphic behaviour was evident from the measurements of light scatter and pulse width distributions. A pattern of high growth yielding smaller cells and less heterogeneous populations could be observed. Analysis of pulse width distributions revealed significant morphological heterogeneities within the bacterial cell population under non-optimal growth conditions, and pointed towards low temperature, high initial pH, and high oxygen levels all being triggers for changes in morphology towards cell chain formation. However, cell size did not correlate to survivability after freeze-thaw or freeze-drying stress, indicating that it is not a key determinant for physical stress tolerance. The fact that *L. reuteri* morphology varies depending on cultivation conditions suggests that it can be used as marker for estimating physiological fitness and responses to its environment.

## Introduction

Probiotics are live microorganisms which when administered in adequate amount confer a health benefit on the host^1^. *Limosilactobacillus reuteri* subsp. *kinnaridis* DSM 17938^2,3^, previously known as *Lactobacillus reuteri* DSM 17938, is one of the most studied probiotic bacterial strains^4,5^, and has been shown to possess several desired probiotic properties^4,6^.

Industrial production of probiotic bacteria must be tailored to reach a stable product with resistance to unfavourable environmental conditions during storage while retaining probiotic functionality^7–9^. Freeze-drying (FD) is one of the most commonly used stabilization processes to achieve probiotic products with a long shelf-life^10^. During FD, cells experience multiple physical stressors that may negatively influence cell viability, for example dehydration, osmotic stress, shear stress and freeze stress^11^. Removal of water results in instability of proteins, DNA and lipids that eventually changes the structure of the cell^11^. The physiological state of the bacteria, including metabolic and energetic state, presence of compatible solutes and fatty acid composition of the cell membrane have previously been found to be key determinants for survival during FD^12–15^. These bacterial properties can be improved by optimising the conditions during cultivation, such as medium composition, pH and temperature^14,16–19^.

Culture conditions causing slow growth, and/or entry into stationary phase have previously been found to increase microbial cell tolerance to FD stress as well as subsequent exposures to multiple other stress factors e.g., gastric acid, or bile^16^. Hernández et al., 2019, demonstrated that high pH (6.5) during fermentation of *L. reuteri* resulted in higher survival rates after FD because of high levels of unsaturated fatty acids, while growing them at low pH (4.5) aided in coping with subsequent acid stress better. Low temperature during cultivation has also been found to correlate to freeze–thaw (FT) stress tolerance, due to an increased proportion of unsaturated fatty acids in the cell membrane^17^. Experiments with *L. reuteri* I500713 and *E. faecium* HL719 showed that preconditioning the cells by including an adaptive step where the temperature was elevated had a positive impact on survival after FD. Studies performed in *L. rhamnosus* GG revealed that cells harvested at late stationary phase grown under uncontrolled pH survived FD better than cells grown under controlled pH^18^. Ampatzoglou et al., 2010 suggest that this might be due to the fact that the cells cultivated in uncontrolled pH environment has already been pre-exposed (preadaptation step) to low pH levels that confers protection to the cells against the low temperature and pressure stresses that follows during freeze drying process.

For *Lactobacillaceae*, cell morphology distributions are often linked to the physiological state of the cell population and hence the cultivation conditions. Cells cultivated at pH 6 was previously found to result in elongated cells for *L. reuteri* when compared to pH 5^14^. For *Lactobacillus acidophilus,* exposure to secondary bile salts (sodium glycodeoxycholate) resulted in elongated and filamentous cells, and inhibition of growth^19^. In contrast, it was reported that for *L. acidophilus* short rods formed as a consequence of suboptimal growth conditions and these cells were significantly more tolerant to FD^20^. Monitoring cell size distributions can thus serve as an indicator of growth deficiencies in fermentation process control, and we hypothesize that it may be useful for the prediction of cell robustness towards subsequent FD stress.

Flow cytometry is a rapid technique to assess multiple cellular properties at the single-cell level by measuring cell fluorescence and light scatter. Fluorescent dyes are used to measure various cell properties such as changes in cellular function, or metabolic activity^21,22^, and light scatter signals can provide valuable information on cell morphology^23^. Flow cytometric fingerprinting is being used for physiological characterization and microbial strain determination^24^. Cell volume was previously shown to correlate to forward scatter (FSC) as well as side scatter (SSC) signals^25–28^. In larger eukaryotic cells, FSC correlates to cell size, while SSC represents cell density or granularity^29^. Another descriptor for cell size recorded in most flow cytometers is the pulse width signal that to date, mostly have been used to discriminate single cells from doublets^30^. Pulse width has, to the best of our knowledge, hitherto not been used as a marker of cell morphology in process analytical technology (PAT) for fermentation processes.

In this study, flow cytometry was applied to assess morphological differences in *L. reuteri* DSM 17938 in response to varying temperature, initial pH, and oxygen levels during cultivation. Multiple cell size descriptors based on light scatter and pulse width measurements were calculated, including subpopulation distributions, arithmetic means, skewness, coefficient of variation (CV) and robust coefficient of variation (rCV). Correlations to bacterial growth, and tolerance to FT and FD stress were also evaluated.

## Materials and methods

### Microorganism and culture conditions

*Limosilactobacillus reuteri* subsp. *kinnaridis* DSM 17938 (previously named *Lactobacillus reuteri* DSM 17938^2,3^), provided by BioGaia AB, was used for all the experiments. A cryopreserved quality-assured cell bank of strain DSM 17938 was created by two-step cultivation in MRS media (Merck, Germany) and suspension in a cryopreservation medium (Supplementary Material, S1-1). The identity of the cell bank was verified by a strain specific PCR using the primers LR1/1694 (5′-TTA AGG ATG CAA ACC CGA AC-3′) and r (5′-CCT TGT CAC CTG GAA CCA CT-3′)^31^. Growth kinetics and vitality were assessed in a BioScreen C® instrument (OY Growth Curves Ab Ltd, Finland), and contamination analysis was performed by cultivation on homofermentative-heterofermentative differential (HHD) agar (Supplementary Material, S1-2). MRS media (Merck, Germany) was used for all the cultivations throughout the work. The pH of the media was adjusted to the desired value by adding 0.1 M NaOH (Merck, Germany) or 1 M HCl (Merck, Germany). For pre-cultivation, a cryo-tube was thawed in a water bath at room temperature for 3 min, and 500 µl of the stock was inoculated to a 50 ml MRS medium (pH 5.86) in a 50 ml Falcon tube. Cells were pre-cultivated without shaking at 37 °C for 16–20 h. The optical density was measured at 620 nm (OD_620_) using a U-1100 spectrophotometer (Hitachi, Tokyo, Japan). Subsequently, the pre-culture was used to inoculate 100 ml of MRS medium to an initial OD_620_ of 0.1, and cultivations were performed in 1L flasks with or without baffles for 24 h. Media and culture conditions were according to the Box–Behnken (BB) design with the three factors temperature (T), initial pH and oxygen at three levels. The BB design was set in MATLAB R2019a using the function *bbdesign*. The order of the experiments (Table 1) was randomised. To generate different oxygen levels, the baffled and non-baffled flasks were either standing still or shaken at 150 RPM, and the gas liquid mass transfer coefficient (k_L_a) was estimated from previously determined values^32^. Temperature was set by incubation in a static incubator (Eppendorf AG, Germany) or a shaking incubator (Termaks AS, Norway). Cell counts were measured by flow cytometry, and cell growth was calculated as the difference in ^10^logarithm of AFU/ml between 0 and 24 h. Cell count at start (0 h) was determined to be 1.74 × 10^7^ ± 1.62 × 10^6^ AFU/ml from ten independent cultures (n = 10).

### Freeze–thaw stress assay

The FT stress assay was performed to investigate the impact of freezing and thawing on the survivability of the cells post-harvest. At the end of each fermentation, the culture was harvested by centrifugation (Eppendorf 5810R, Germany) at 3000×*g* for 10 min. The supernatant was carefully discarded and the cells were concentrated 20-fold by resuspending the cell pellet in phosphate buffer saline (PBS) (Merck, Germany). An equivalent volume of PBS containing 20% (w/v) sucrose (Acros organics, Belgium) was added, thus reaching a final sucrose concentration of 10%. Cell samples were frozen at − 80 °C for 5–10 days, and carefully thawed under controlled conditions by incubating the sample vials in a water bath at room temperature for 15 min. Cells were subsequently used directly for further analysis by flow cytometry.

### Freeze-drying procedure

Apart from the 15 cultivations generated by BB design, three additional cultivations were carried out to evaluate FD stress. The three cultivations were chosen based on the results of BB design experiments and were performed in biological duplicates. Bacteria were cultured under the conditions found to cause variation in size distributions and at the same time enable growth (T = 30, 37 and 44 °C, pH 6, kLa [O2] 0 h^−1^). Cells were pre-formulated in PBS with 10% sucrose as described above for the freeze–thaw assay. Before freezing, cell suspensions were transferred to 2 ml aliquots in 8 ml Schott FD vials, and the vials were covered with a rubber cap. Samples were pre-frozen in an -80 ˚C freezer where the samples were stored for 24–36 h before further processing. FD was performed with Epsilon 2-6D LSCplus (Martin Christ, Germany). Frozen samples (− 80 °C) were transferred rapidly to pre-cooled shelves (− 40 °C) in the freeze dryer and allowed to acclimatize for 2 h. Primary drying was started by lowering the pressure to 0.072 mbar measured with a Pirani gauge (equivalent to − 45 °C in vapour pressure) and the shelf temperature was increased to – 38 °C with a rate of 0.13 °C/min. The end of the primary drying was determined to be the point when the capacitance pressure reached the Pirani pressure. Subsequently, the secondary drying was carried out at the same chamber pressure as in primary drying and the shelf temperature was increased to 20 °C with a rate of 0.083 °C/min. Freeze-dried samples were sealed under vacuum and stored at − 80 °C. Before analysis, the freeze-dried samples were rehydrated in 2 ml sterile deionized water at room temperature. The entire chain of steps including freezing, drying and rehydration is referred to as FD stress.

### Microscopic determination of cell volume

Microscopic pictures were captured using Lecia DM500 microscope equipped with a 40 × Hi Plan Microscope Objective (Leica Microsystems, Switzerland) according to the manufacturer’s instructions. Cell samples were centrifuged at 3000×*g* for 2 min and resuspended in PBS to obtain an OD_620_ between 5 and 6. Five µl sample was drawn and placed on the glass slide which was then covered by the coverslip to examine under the microscope. Phase contrast tray was used to enhance the contrast of the microscopic images as they were colourless (no staining). The software ImageJ 1.52n^33^ was used to determine the length and width of individual cells for the microscopic images. Cell volume was calculated using the length and width of the cell, assuming cells to have a cylinder shape^25^. Further details on the microscopic analysis are provided as supplementary information (Supplementary Material, S3).

### Scanning electron microscopy (SEM) imaging

Freeze-dried bacterial samples were thawed in 2 ml of MilliQ water and washed twice by centrifugation at 3000×*g* for 5 min. Two drops of each sample were dropped on an aluminium stub with carbon tape and allowed to dry under a light for 2 h. After drying the samples, the samples were sputter coated twice with gold at 0.1 bar for a total of 60 s. Micrographs were acquired in a FlexSEM 1000 II scanning electron microscope (Hitachi, Tokyo, Japan), using the imaging software FlexSEM. Post-imaging analysis was performed in Affinity Photo v 1.8.4.

### Flow cytometry

A BD Accuri C6 plus flow cytometer was used to record cell fluorescence, forward scatter (FSC), side scatter (SSC), and pulse width. Concentrations for fluorescent dye staining for determination of cell viability was as described previously^16^. In brief, cell samples were centrifuged at 3000×*g* for 2 min and resuspended in PBS to an OD_620_ of 0.01–0.05. Thereafter a mix of SYBR® Green I (Thermofisher Scientific, Sweden) and Propidium Iodide (PI) (Sigma-Aldrich, Germany) were added to 0.5 ml samples at a concentration of 5 µg/l of 100X SYBR® Green I and 1 µg/l PI. Samples were vortexed, incubated in darkness for 15 min at 37 °C and vortexed again and then used directly for analysis. Excitation wavelength for the laser used was 488 nm. Fluorescence emission levels were measured using a band pass filter at 530/30 nm (FL1, SYBR® Green I) and a long pass filter at > 670 nm (FL3, PI). A sample volume of 25 or 50 µl was collected with a flow rate set to medium (35 μl/min), and a threshold of 500 channel number (chnr) on FL1-H (533 ± 30 nm), and no threshold on FL3-H (> 670 nm) for recording SYBR® Green I and PI fluorescence, was applied. Data were exported as FCS2.0 files and processed using the FlowJo v10 software (FlowJo, LLC, USA). Gating strategy was performed based on previous work by Nescerecka et al., 2016 where the cells were gated into intact and damaged cells^34^. Flow cytometry results for intact cell concentrations were expressed as Active Fluorescent Unit (AFU)/ml. AFU can be considered as intact cells able to replicate plus cells that retain an intact cell membrane^35^. Distribution of FSC-H and SSC-H were used to calculate the arithmetic mean, mode, median, coefficient of variation (CV), robust coefficient of variation (rCV), as described previously^36^. Skewness is the measure of distortion from the normal distribution within a dataset. A normally distributed data will have zero skewness. If the skewness value obtained is positive, it is right skew and if the skewness is negative it is left skew^36^. Pulse width subpopulations were gated based on *create gate on peaks* function on FlowJo v10 software. The gates 1 and 5 were manually adjusted to have full coverage without changing the gates with high cell distributions (2, 3 and 5). Further details on the flow cytometry analysis are provided as supplementary information (Supplementary Material, S4).

### Statistical data analysis

Statistical analysis was used to describe the effects of intrinsic factors (T, initial pH, and oxygen concentration) on the following output variables: cell growth (Δ log AFU/ml), FT survivability (%), and multiple variables derived from flow cytometry measurement of light scatter and pulse width distributions. Correlations between output variables were also analysed. Graphpad Prism v 9.2.0 built-in analysis of Pearson’s correlation was used to evaluate the effect of each factor on the output variable. The variables were assigned with a correlation coefficient r value and a *p*-value < 0.05 was considered statistically significant. A three-variable Box–Behnken design with three replicates at the centre point was selected to build the response surface models. The design was used to determine an optimum for cell robustness against various environmental factors by fitting the polynomial model based on the response surface methodology by using the toolbox *curve fitting* on MATLAB 2019b.

## Results

### Effect of temperature, initial pH, and oxygen on cell growth, and viability

To investigate effects of the process variables temperature (T), initial pH and oxygen on growth, viability, and cell morphology, cells were cultured under different conditions according to a BB design of experiments (Table 1). The choice of BB design was motivated by the possibility to find significant correlations between variables within a rather limited number of experiments^37^. Fifteen cultivations were performed, whereof three are biological triplicates of the central point which gave information of the batch-to-batch variation. Output variables Δ log AFU/ml, viability (%) had < 5% of variation between the triplicates. Levels of specific input variables (T, initial pH, oxygen) were set towards conditions that were suboptimal for growth, to study if moderate to severe stress conditions could trigger increased cell robustness, as well as rearrangements in cell morphology.

**Table 1:** Box–Behnken design of experiments generated by MATLAB R2019a using the function *bbdesign* with three variables and three levels.

Condition	Temperature (°C)	Initial pH	k_L_a (h^−1^)
1	30	5	47.5
2	30	7	47.5
3	44	5	47.5
4	44	7	47.5
5	30	6	0
6	30	6	74.4
7	44	6	0
8	44	6	74.4
9	37	5	0
10	37	5	74.4
11	37	7	0
12	37	7	74.4
13	37	6	47.5
14	37	6	47.5
15	37	6	47.5

**Figure 1:**
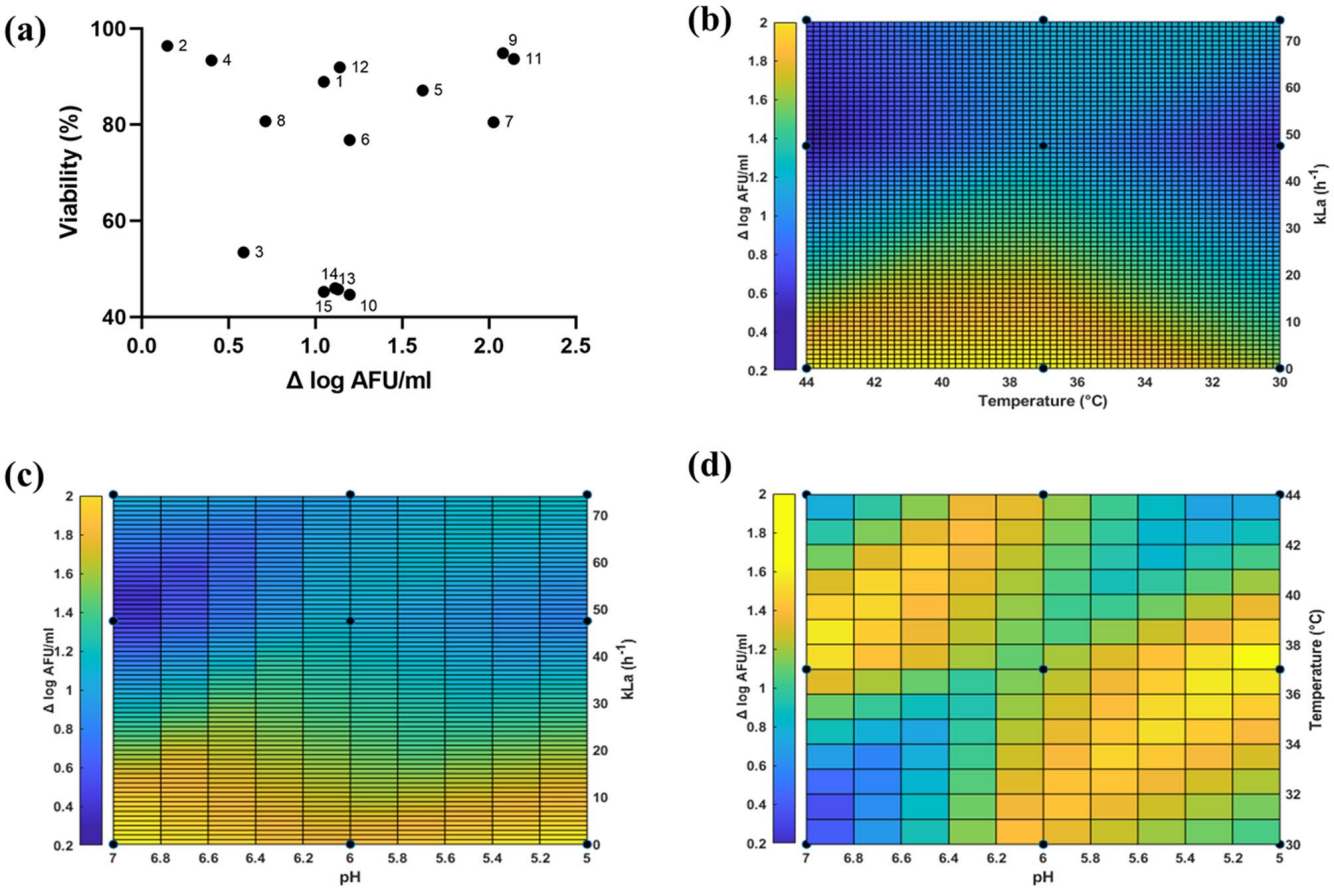
Growth and Viability. Viability is defined as the ratio of intact cells and total cells (**a**) Evaluation of viability (%) as a function of growth (Δ log AFU/ml). The graph indicates 3 clusters indicating viability is independent of growth (Correlation coefficient r = 0.3723, p-value > 0.05). Surface plots of (**b**) pH and oxygen rate (kLa (h^−1^)) (**c**) Temperature and oxygen rate (kLa (h^−1^)), (**d**) pH and temperature against growth (Δ log AFU/ml). The intensity is displayed by the scale bar, blue colour denotes low growth (Δ log AFU/ml) and yellow colour denotes high growth (Δ log AFU/ml).

As expected, a large variation in growth behaviour and viability was observed for the different conditions evaluated (Fig. 1a). Viability is defined as the ratio between intact cells and total cells, in percent. Growth ranged from 0.146 Δ log AFU/ml (condition 2) to 2.143 Δ log AFU/ml (condition 11), and viability varied from 44.6% (condition 10) to 96.4% (condition 2). A considerable number of cultures displayed low growth, but this was not correlated to poor viability since most cultures displayed viability > 80% (conditions 1, 2, 4, 5, 7, 8, 9, 11, 12). A factor in common for cultures with high Δ log AFU/ml (conditions 5, 7, 9, and 11) was that they were exposed to negligible levels of oxygen, although temperature and initial pH also seemed to play a role (Fig. 1b–d). Oxygen concentration exhibited a moderate negative correlation with r-value of − 0.6846 and p-value of 0.0049, which indicated it is significant for growth (Table 2), while temperature and initial pH was not found to have a significant influence under the investigated conditions. As for viability, oxygen concentration and initial pH displayed low correlation (r-value = − 0.3592 and 0.4437 respectively) and temperature had negligible correlation and all three variables had p-value > 0.05 (Table 2).

**Table 2:** Impact of temperature, pH and oxygen concentration (kLa) on output variables.

Output variable	Pearson correlation coefficient r			p-value		
	Temperature	Initial pH	kLa (h^−1^)	Temperature	Initial pH	kLa (h^−1^)
Δ log AFU/ml	− 0.04535	− 0.1721	− 0.6846	0.8725	0.5398	0.0049
Viability	− 0.1690	0.4437	− 0.3592	0.5679	0.1069	0.2005
FSC-H	− 0.1388	0.4694	0.3671	0.6217	0.0775	0.1783
SSC-H	− 0.3603	0.4245	0.173	0.1871	0.1148	0.5374
FT survivability	− 0.387	− 0.6052	− 0.1169	0.1541	0.0168	0.6783

*FSC-H* Forward scatter height, *SSC-H* Side scatter height.

### Impact of culture conditions on cell morphology and correlations to growth

**Figure 2:**
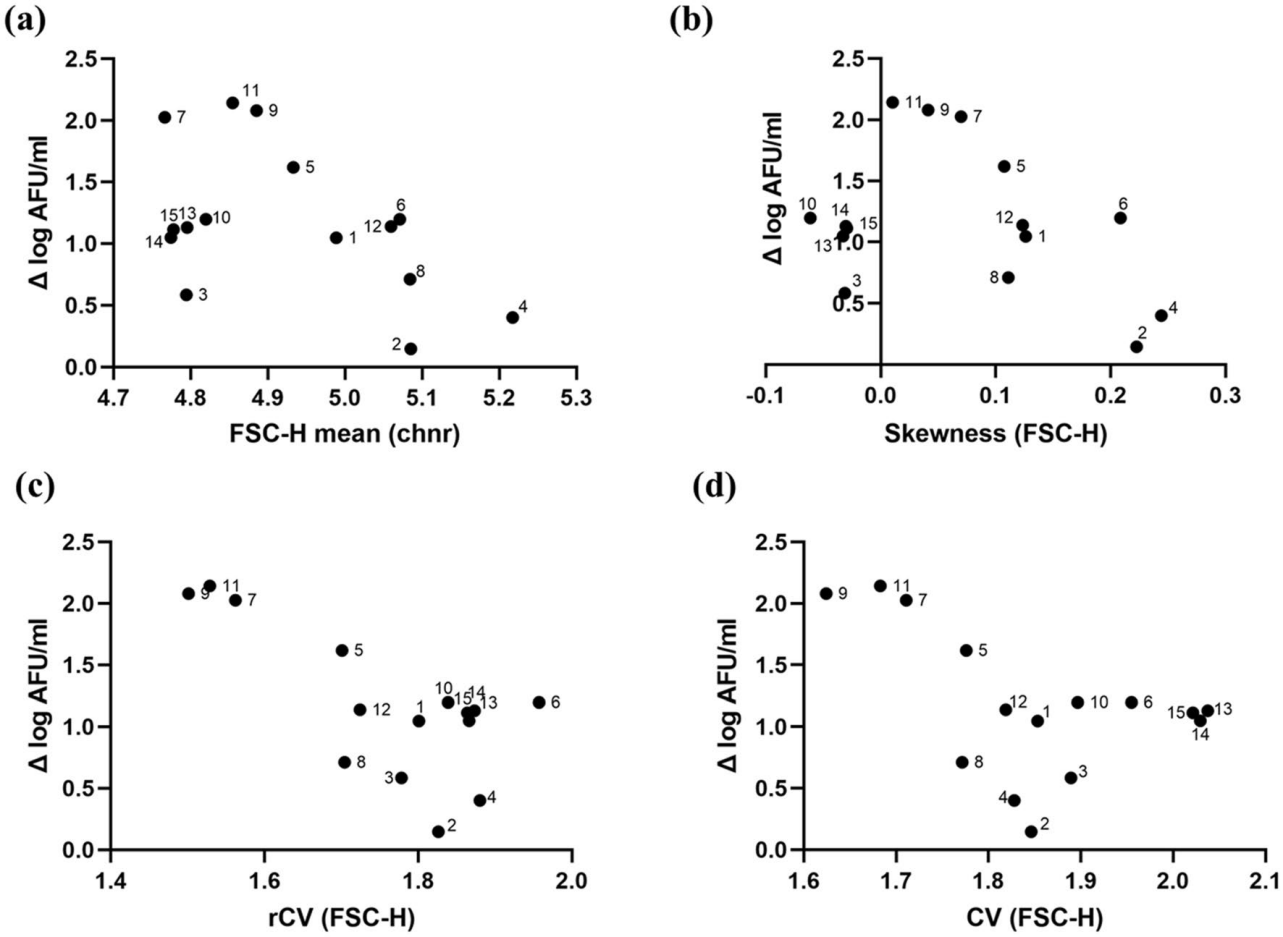
Morphology descriptors calculated for 15 conditions (**a**) FSC-H mean, (**b**) Skewness of FSC-H, (**c**) rCV of FSC-H, (**d**) CV of FSC-H vs growth (Δ log AFU/ml). Chnr-channel number.

Bacterial cell morphology was analysed by light microscopy and flow cytometric light scatter data. The arithmetic means of cell volume (Supplementary Material, Table S3-1), FSC-H and SSC-H (Supplementary Material, Table S4-1) pointed towards that cell morphology was highly variable under the different conditions, with no significant correlation to growth or with any of the three process variables (Table 2). However, there was a tendency towards higher mean FSC-H for cultures displaying poor growth (Fig. 2a). Large values did not coincide with high Δ log AFU/ml under any condition evaluated, which suggests that a considerable proportion of large cells is a sign of poor growth. It should be highlighted though that there was only a weak correlation between cell volume obtained from the microscopic analysis and means of FSC-H and SSC-H (Supplementary Material, Fig. S4-2a,b), demonstrating that they are not reporting on identical cell parameters, e.g., light scatter also depends on difference in refractive index between cells and the surrounding liquid^29^.

The mean FSC-H varied between 4.7 chnr and 5.2 chnr and the highest growth was observed for values between 4.75 chnr and 4.90 chnr (Fig. 2a). Similarly, SSC-H means varied between 3.95 chnr and 4.3 chnr, and the highest bacterial growth was observed at a value of 4.05 chnr (Supplementary Material, Fig. S4-3b). FSC-H and SSC-H displayed low to negligible correlation and yielded p-value > 0.05 (Table 2) for three process variables. Batch variation was based on the triplicates of the central point and displayed high variability for mean cell volume (24.6%) and very low variability for FSC-H and SSC-H (0.2% and 0.4% respectively).

**Figure 3:**
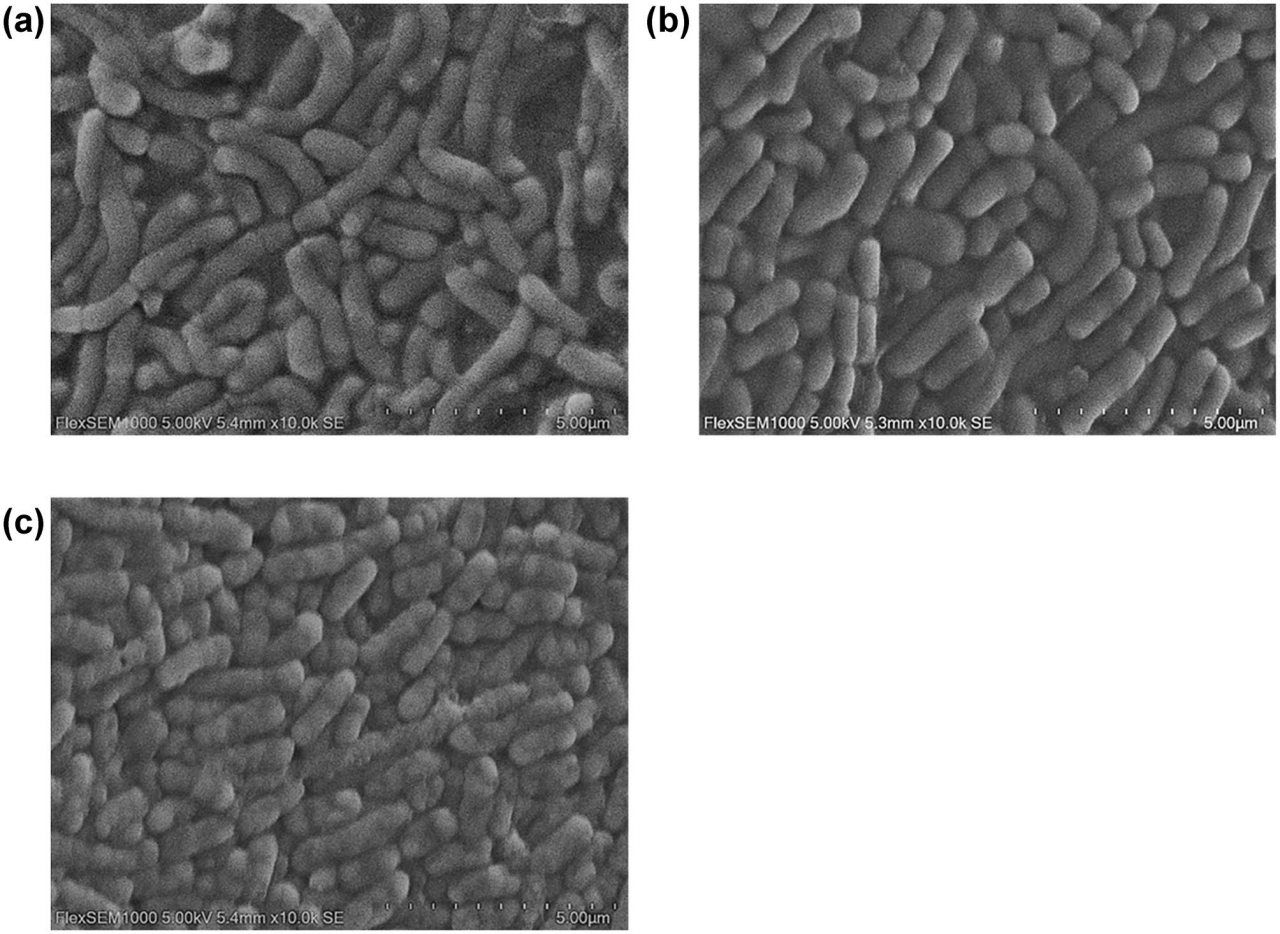
Scanning Electron Microscopy imaging of cells cultivated at (**a**) 30 °C, (**b**) 37 °C and (**c**) 44 °C.

Scanning electron microscopy (SEM) was also carried out on three representative cultures with bacteria having different cell size distributions but with similar growth (grown at 30 °C, 37 °C, and 44 °C) (Fig. 3). The SEM analysis revealed that the larger bacteria obtained from the 30 °C cultivation consisted of chains with two or more cells attached together, with clear septa (Fig. 3a). Cultures grown at 37 °C and 44 °C consisted of cells with smaller size when examined carefully by visual inspection.

### Assessment of population heterogeneity by light scatter and pulse width distributions

The degree of population heterogeneity was quantified by analysing FSC-H and pulse width distributions, which varied highly between cultures. FSC-H distributions did not appear to consist of separable subpopulations with different morphology, so the degree of heterogeneity was assessed by calculating skewness, coefficient of variation (CV), and robust CV (rCV), as described previously^36^ (Fig. 2; Supplementary Material, Table S4-1). Both left and right skews were observed, with a range from − 0.06 to + 0.22 and a tendency towards normal distribution (+ 0.02 to + 0.05) for cultures displaying high Δ log AFU/ml. Similarly, low CV (1.62 to 1.71) and low rCV (1.5 to 1.56), indicating less heterogeneous populations, also coincided with good growth, while the values were higher for cells with poor growth (Fig. 2c,d). This tendency was significantly more pronounced for rCV compared to the mean FSC-H described above. rCV displayed high correlation with r value = − 0.7137, p-value = 0.0028, whereas CV and skewness displayed low correlation (r value = − 0.4968 and − 0.3601 respectively) with p-value > 0.05 (Supplementary Material, Table S4-3).

**Figure 4:**
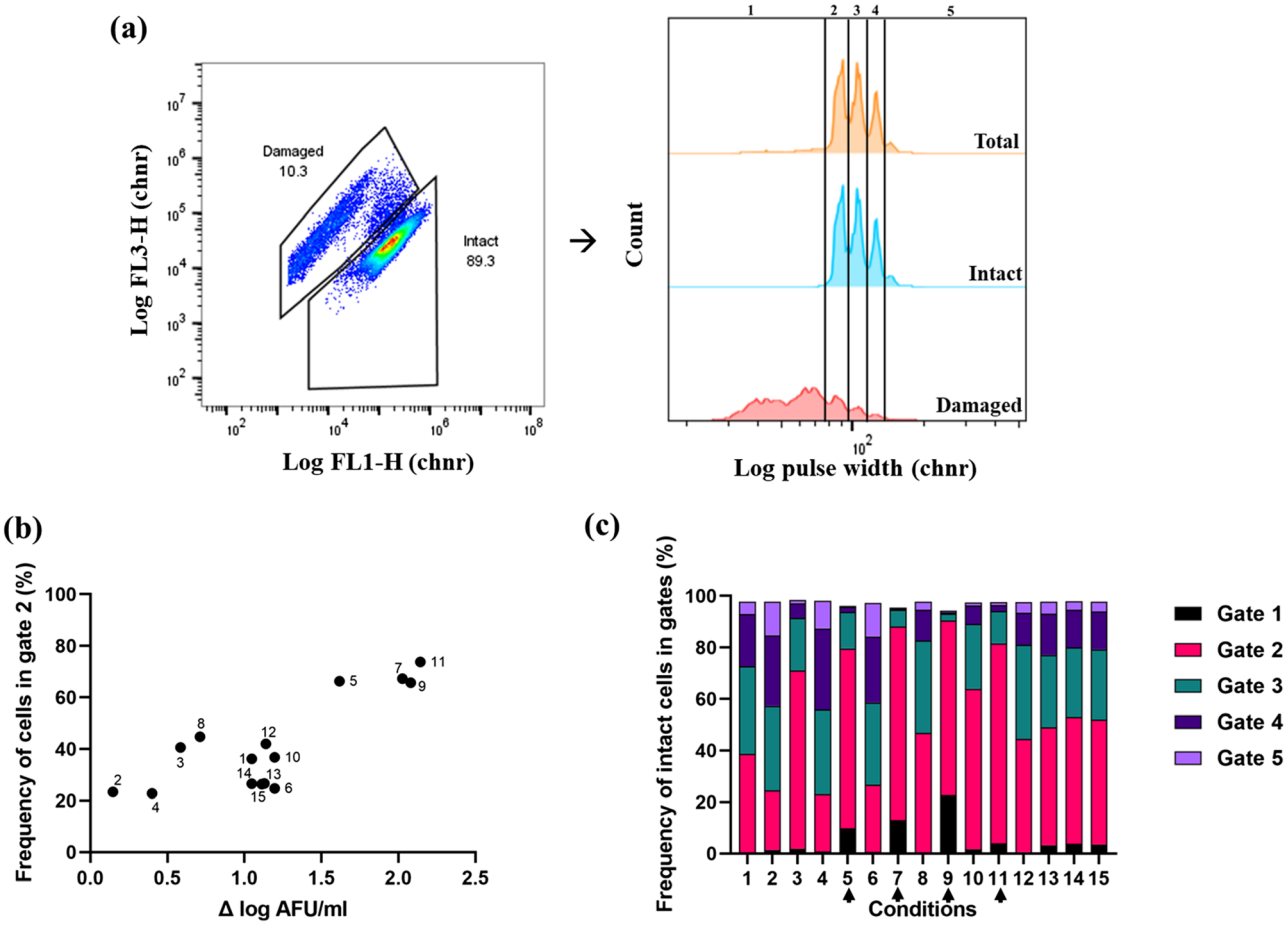
Pulse width distribution. (**a**) Representation of pulse width distribution and gating strategy to quantify frequency of cells in gates 1–5 for condition 6. Gates were defined using the built-in function in the FlowJo software *Create Gates on Peaks* (Supplementary Material, Fig. S4-2). The black lines indicate the different gates. (**b**) Correlation between frequency of cells in gate 2 (%), and growth (Δ log AFU/ml). (**c**) Distribution of intact cells in gates 1–5 based on pulse width peaks for conditions 1–15. Black arrow indicates the conditions with high Δ log AFU/ml.

In contrast to FSC-H, the pulse width distributions appeared as distinct subpopulations that were classified with five separate gates (Fig. 4a). Pulse width correlates to the time it takes for a cell to pass the laser interrogation point and provides information of its size^30,38,39^. Previous reports have stated that there is a tendency for cells to align along their long-axis by the hydrodynamic focusing of the cytometer^40–42^. With support of this previous work, the separation into distinct subpopulations may indicate chains with a varying number of uniform cells with distinct lengths. From the SEM analysis the presence of both single cells and chains with a different number of cells, and more of the latter at 30 °C compared to 37 °C was observed (Fig. 3).

The frequency of cells within each of the five gates (Fig. 4a) was calculated for the total population, as well as for intact and damaged cell subpopulations. The frequency of total cells in gate 2 correlated well with the bacterial growth (Δ log AFU/ml) (Fig. 4b), (r = 0.8083 and p-value = 0.0003). The cultivations with strong growth (conditions 7, 9 and 11) had above 60% of the cells in gate 2 suggesting that the population is less heterogeneous which is similar to other heterogeneity variables determined in the study (CV, rCV and skewness).

Intact and damaged cells displayed a significant difference in subpopulation distribution. A paired *t*-test highlighted the significance between damaged and intact cell populations in gate 1 and 2 with p*-*values < 0.0001. The damaged cell subpopulations predominantly ended up in gate 1 for all the cultivations (> 40%), while the living cell had the majority in gate 2 (> 40%) for all cultivations except for conditions 2, 4 and 6 (Supplementary Material, Table S4-4). The frequency of intact cells in gate 2 was found to depend on culture condition (Fig. 4c).

Surface plots of frequency of cells in gate 3–5 were made to visualise which conditions resulted in larger cells (Supplementary Material, Fig. S4-6). High oxygen levels, high initial pH, and low temperature resulted in larger bacteria, possibly by acting as triggers for chain formation, which was inversely correlated to cell growth. From the correlation coefficient analysis, oxygen concentration had a high positive correlation r = 0.7406 and significant impact on pulse width distribution (frequency of cells in gates 3–5) (p*-*value = 0.0016). Temperature and initial pH had negligible to low correlation with a r-value of − 0.2355 and 0.3766 respectively with p-value > 0.05. Batch variation was based on the triplicates of the central point and displayed low variability for CV and rCV (< 1%) and skewness (5.5%). As for pulse width distribution gate 1–3 consisted > 80% of the total cells (except for condition 2 and 4) displayed low variability (< 5%) and gate 4 and 5 which consisted ≤ 20% of total cells displayed much higher variability (> 5%).

### Tolerance to freeze–thaw stress

**Figure 5:**
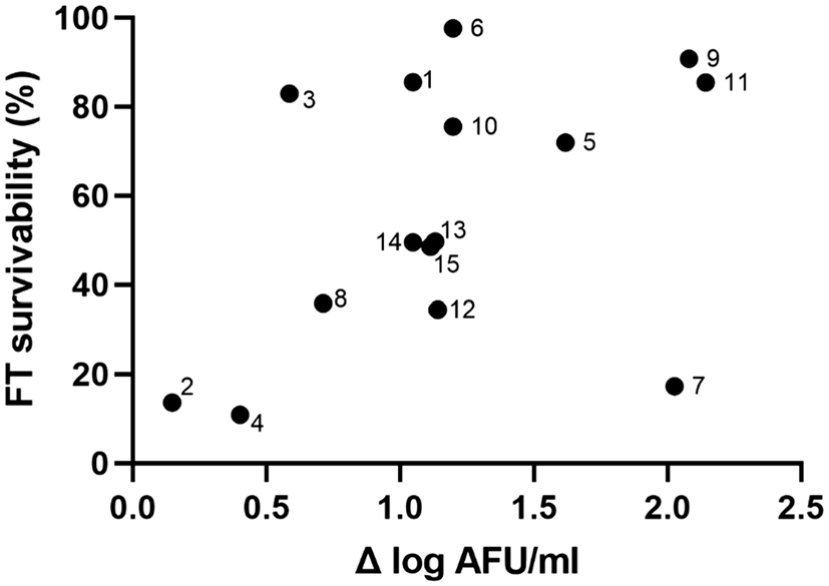
Freeze–thaw (FT) survivability (%) plotted against growth (Δ log AFU/ml). FT survivability (%) is calculated as a ratio of viability of cells after FT stress to viability of cells at harvest. (Correlation coefficient r = 0.4291, p-value > 0.05).

To investigate differences in FT stress tolerance amongst the cultures, cells were subjected to one cycle of controlled freezing and thawing, and the survivability was then determined. FT survivability is defined as ratio between intact cells after and before FT stress. A large variation in survivability was observed spanning from 10.9 to 97.6%. There was negligible to low correlation to cell growth (Δ log AFU/ml) with r-value = 0.4291, p-value > 0.05) (Fig. 5) or to any of the cell morphology descriptors (Supplementary Material, Fig. S5-1, Table S5-1). High survivability was seen for bacteria cultivated under sub-optimal conditions (condition 6), as well as for several of the cell cultures with low cell counts. Overall, the results suggested that slow growing bacteria are not per se robust to FT stress; instead, the specific combination of environmental factors play the dominant role. Slower growth due to the presence of high oxygen levels seemed to make the cell populations more tolerant to FT stress, although only at pH ≤ 6, and at temperatures suboptimal for growth (condition 1, 3, and 6). Pearson correlation analysis indicated that initial pH had a moderate negative correlation (r-value = − 0.6052) to FT survivability yielding p-value = 0.0168. Temperature and oxygen concentration displayed negligible to low correlation (r-value = − 0.3870 and − 0.1169 respectively) yielding p-value > 0.05. FT survivability displayed low variation (< 5%) for the central point (triplicates) (Supplementary Material, Fig. S2-2).

### FD stress tolerance of subpopulations with distinct morphologies

**Figure 6:**
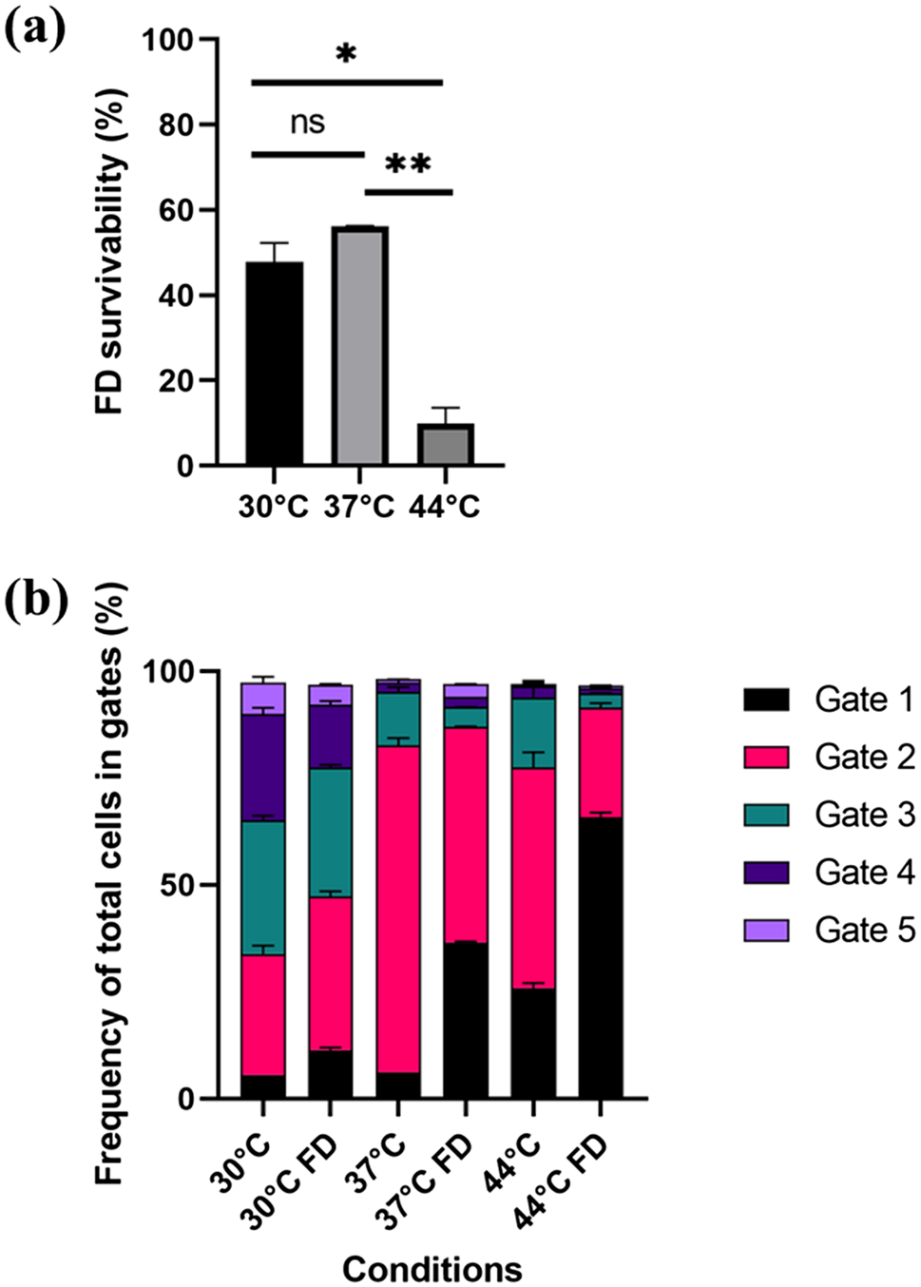
(**a**) Freeze-drying (FD) survivability (%) for the cells cultivated at 30 °C, 37 °C and 44 °C. Statistical significance was calculated using student t-test: ns = not significant, * indicates *p*-value < 0.05, **indicates *p*-value < 0.01. Data are mean and SD of two biological replicates for 3 conditions. (**b**) Pulse width distribution (frequency of cells in each gate 1–5) for total cells before and after freeze-drying stress.

The impact of FD stress on bacterial cells as categorised by morphology descriptors was studied. Bacteria were cultured under three different conditions found to cause variation in size distributions and at the same time enable growth (T = 30, 37 and 44 °C, pH 6, kLa [O_2_] 0 h^−1^). Final cell titers after 24 h cultivations were 8.44 × 10^8^ ± 2.35 × 10^8^ AFU/ml (30 °C), 5.40 × 10^8^ ± 9.19 × 10^7^ AFU/ml (44 °C), and 2.13 × 10^9^ ± 6.36 × 10^7^ AFU/ml (37 °C). Freeze-drying survivability was higher for cells grown at 37 °C (56.1% ± 0.24%) and 30 °C (47.87% ± 4.41%) than for cells grown at 44 °C (9.96% ± 3.64%) (Fig. 6a).

Microscopic analysis showed that cultivation at 37 °C resulted in smallest cells (mean cell volume ≈ 1.79 ± 0.22 µm^3^), followed by 44 °C (mean cell volume ≈ 3.14 ± 0.83 µm^3^), and the largest at 30 °C (cell volume ≈ 3.62 ± 0.39 µm^3^). Mean cell volume was significantly different between 37 and 30 °C which yielded a p-value = 0.0288. For bacteria grown at 30 °C, there was a relatively even distribution of viable cells in pulse width gates 2–4 (28.50% ± 1.91%, 31.25% ± 1.06% and 24.80% ± 1.56% respectively) (Fig. 6b). This was different from bacteria grown at 37 °C and 44 °C, where a majority of the cells were found in gate 2 (76.55% ± 1.63%, and 51.60% ± 3.54, respectively). FD treatment changed the pulse width distributions to a significant degree for the total cell subpopulations, with a higher frequency of cells in gate 1 after FD (Supplementary Material, Table S4-6) (Fig. 6b). This difference might be due to the fact that FD increase the number of damaged bacteria, which ends up gate 1 and 2. For intact cell subpopulations, there was no significant difference in pulse width distribution before and after FD stress indicating that FD stress did not cause a reduction in the size of the bacteria, for example by chain disruption.

## Discussion

Bacterial morphology varies depending on conditions applied during fermentation and may be used as a marker for estimating physiological fitness and responses to its environment. A recurring consequence of suboptimal process conditions for several pleomorphic bacteria is a shift towards larger cell sizes or chain formation^20^. In this study, flow cytometric measurement of light scatter and pulse width distributions revealed a pronounced pleomorphic behaviour of *L. reuteri* DSM 17938 when exposed to different temperature, initial pH, and oxygen. All the FCM variables (means, skewness, CV and rCV of FSC-H and SSC-H), as well as a gating strategy based on pulse width distributions to describe morphological heterogeneity pointed in the same direction. Cultures reaching high cell densities consisted of less heterogeneous populations of smaller bacteria, while cultures exposed to suboptimal conditions for growth had a large variation in cell morphology. However, light scatter measurements by flow cytometry are not solely dependent on cell size. Other factors such as surface roughness and differences in refractive index between the liquid phase and the cells may also impact the scattering profiles, as discussed previously^29^. Furthermore, the instrument design for FSC measurements typically differs amongst flow cytometers from different manufacturers, and a validation of the present method to describe cell size distributions in *L. reuteri* is required for other instruments.

Slower bacterial growth was observed by lowering of the temperature to 30 °C, regardless of pH and oxygen levels, and coincided with chain formation and a 2–4 orders of magnitude higher mean cell size and population heterogeneity. This was obvious from both SEM analysis, as well as the measurement of pulse width distributions. Likewise, cultures exposed to high levels of oxygen in combination with a higher initial pH displayed a high frequency of larger cells. For *L. reuteri*, it has previously been found by microscopic analysis that a higher pH (6.0 compared to 5.0) during cultivation results in larger cells^14^. In the current study, oxygen or pH 7.0 alone did not result in larger cells for cultures incubated at 37 °C. The correlation between cell size and growth may be attributed to the complex protein ring assembly (e.g., encoded by the FtsZ gene) involved in septa formation during cell division, as chromosome segregation and cell division is not coupled in bacteria. The response levels of the Ftsz gene varies based on the culture conditions as seen in *B. subtilis*^43^ and *L. plantarum*^44^. This suggests that cell morphology is dependent on culture conditions which may result in rapid or slow growing cells on activation or deactivation of the FtsZ gene.

No obvious link between cell morphology and survivability after FT or FD stress was observed in the current study, neither when comparing different cultures nor when comparing subpopulations within the same culture based on pulse width distributions. This demonstrates that it is rather the specific physiological state of the bacterium, as ruled by environmental conditions, that matter^12–14,18,45^. Initial pH alone had a profound effect on cell survivability after FT stress, while oxygen had the highest impact on growth. The negative effect of oxygen may be due to the formation of reactive oxygen species (ROS) such as superoxide radicals and hydrogen peroxide (H_2_O_2_)^46^. However, four out of seven conditions that had survival rates above 70% were with shaking (i.e., aerated with oxygen), showing that beneficial influence of other factors, such as low pH or low temperature, may compensate for the negative effect of oxygen. Effect of initial pH on FT survivability varied across the conditions, but pH 5 being most predominant for robust cultures. A previous study from Palmfeldt and Hahn-Hagerdal 2000, highlighted that growing of *L. reuteri* at different pH (5 and 6) resulted in the same metabolite formations which suggest that the primary metabolism was identical. Before taking into account the effect of pH on the growth, it should be noted that it has not been controlled during the cultivations as the experiments were performed in MRS medium in a shake flask. This ultimately led to the same levels of pH for cultivations having obtained good growth. Therefore, the drop in pH levels might be equivalent in each condition and the cells cultivated at pH 5 are already preconditioned to acidic conditions. Hernández et al. showed that cells growing at varying pH but at a temperature optimum (37 °C) had very similar cell density at harvest. It holds valid with this study as cells cultivated at pH 5 and 7 and at 37 °C yielded equally high cell densities. Lowering the temperature below the optimum of growth confers modifications in fatty acid composition by increasing the membrane fluidity which could have resulted in high viability after FT. An increase in the ratio of unsaturated fatty acids to saturated fatty acids in the cell membrane is an important strategy for adaptation to cold temperature and survival in unfavourable conditions^47,48^. In line with this is that bacteria grown at 44 °C had poor robustness to the FT and FD stress, as well as poor viability after 24 h even though they had high cell density. This could be supported by previously reported growth optima for members of the genus *Lactobacillus* and *Limosilactobacillus* to be between 30 and 40 °C^12^.

## Conclusion

In this study, a FCM pipeline for analysing and correlating environmental factors and cell morphology of *L. reuteri* DSM 17938 during cultivation and subsequent FD processing was established. The FCM-based output parameters were able to capture subtle differences amongst cultures at the subpopulation level and may have potential to be use as PAT tool in process control of morphology during fermentation. In particular, the pulse width data was found useful to monitor the emergence of larger cells, as well as their structural rigidity and survivability when exposed to physical stress treatment. A high robustness of *L. reuteri* DSM 17938 towards FD stress coincided with good growth and a cell population consisting of smaller cell and a narrow size distribution. Although, several cultures prepared under suboptimal conditions resulting in larger cells and heterogeneous size distributions were also found to be robust, demonstrating that cell morphology descriptors alone are poor indicators of FT and FD stress tolerance for this bacterium. Instead, the specific combination of temperature, initial pH and oxygen (different levels) determined the FD tolerance of the culture.

## Supplementary Information

Supplementary Information.
