# Supplementary material for: Flow cytometric analysis reveals culture condition dependent variations in phenotypic heterogeneity of Limosilactobacillus reuteri
Source: Sci Rep. 2021 Dec 7;11:23567. doi: 10.1038/s41598-021-02919-3 (PMC8651721; doi:10.1038/s41598-021-02919-3)
Supplement: Supplementary file 1 — Supplementary Information. [file 41598_2021_2919_MOESM1_ESM.docx]

**Supplementary material:**

**S1.** Media composition

**S2.** OD values for 15 conditions at 24h, Batch to batch variation for the central point condition

**S3.** Microscopic analysis of cell size

**S4.** Flow cytometry analysis

**S5.** Evaluation of correlations between morphology descriptors and FT survivability

**S1. Media composition**

**S1-1 – Cryopreservation medium**

0.82 g K_2_HPO_4_ (VWR chemicals, USA), 0.18 g KH_2_PO_4_ (LabKem, Spain),0.59 g Na-citrate dihydrate (Merck, Germany), 0.25 g MgSO4 x 7 H_2_O (Merck, Germany), 172 ml glycerol (87%) (Merck, Germany) 15% final concentration, Add dH_2_O to 1000 mL

**S1-2 – Homofermentative heterofermentative differential (HHD) agar**

2.5 g Fructose (Sigma Aldrich, USA), 2.5 g KH_2_PO_4_ (LabKem, Spain), 10.0 g Tryptone (Sigma Aldrich, USA), 1.5 g Soy Peptone (VWR Chemicals, USA), 3.0 g Casamino Acids (BD Biosciences, USA), 10.0 g Yeast Extract (Sigma Aldrich, USA), 1.0 g Tween 80 (Merck, Germany), 20 mL Bromo Cresol Green (Sigma Aldrich, USA) dissolved in 0,01N NaOH (Merck, Germany) (0.13 g in 40 mL NaOH), 20.0 g Agar (VWR Chemical, USA), 1 L dH2O, pH correction at 50°C to the value of 6,85 + 0,05, using NaOH 35% (Merck, Germany). Sterilization by autoclavation at 121°C for 15 min.

**Supplementary material S2. OD values for 15 conditions at 24h**


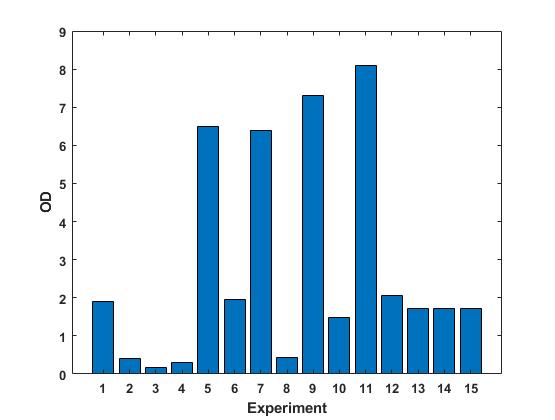


**Figure S2-1.** OD_620_ at 24h for conditions 1-15. Cells were cultivated for 24h in MRS under different conditions.


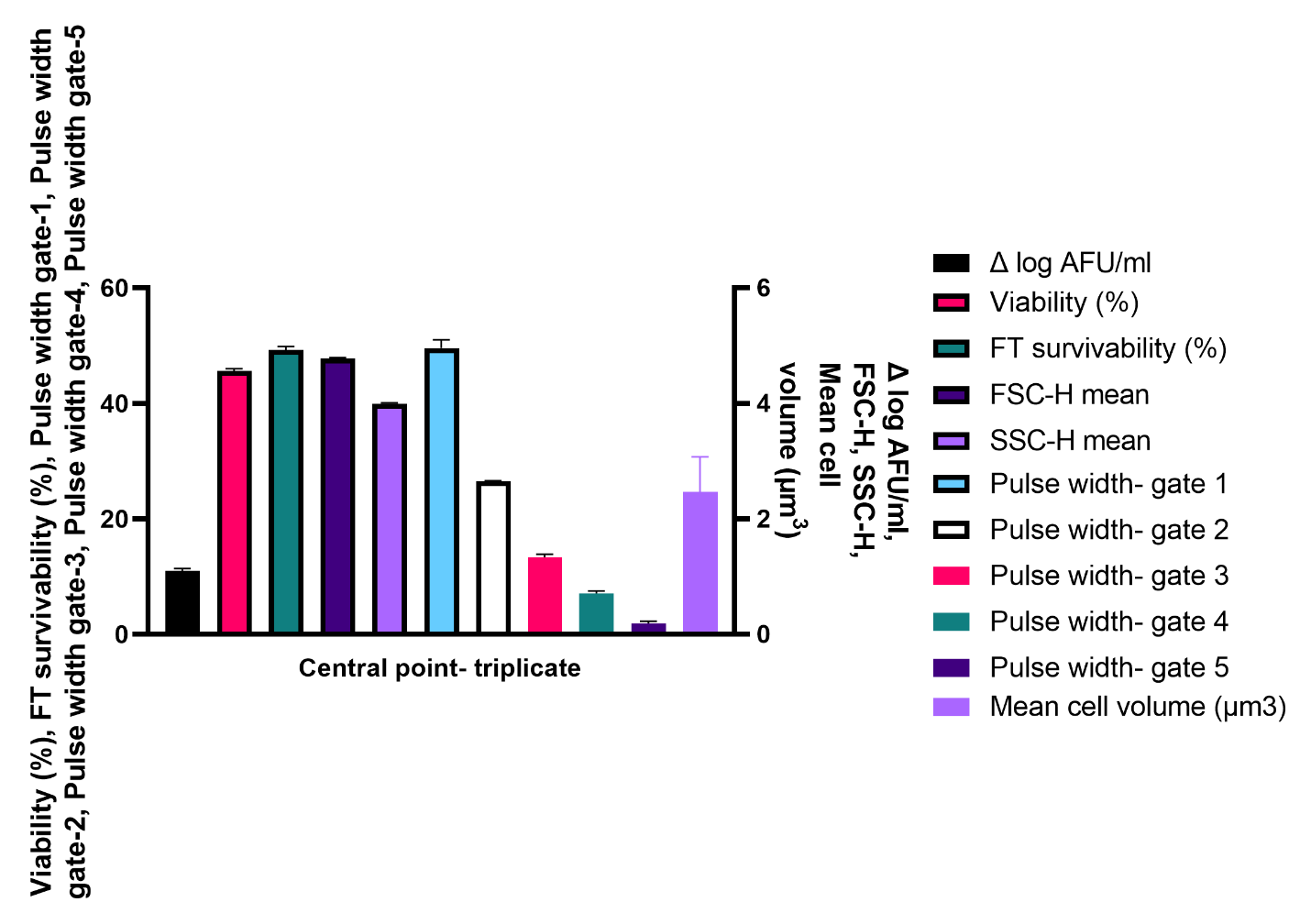


**Figure S2-2.** Batch variation for the central point (condition 13, 14 and 15) in Box Behnken design (n=3).

**Supplementary material S3. Microscopic analysis of cell size**

All the pictures were captured at 40X magnification with a phase contrast light microscope Leica DM750 (Leica Microsystems, Switzerland) integrated with phase turret condenser and phase contrast rings for each magnification. The pictures were captured with automatic exposure. Once the images were obtained, image processing was performed using free software package NIH ImageJ 1.52n. The first step is to take a picture of the scale was taken at 40X. This image is used for processing the length standard-length measuring unit such as µm. By using a straight-line tool, a known length of 100 µm was chosen. Then, a module named set scale was used where the conversion was made. Picture to be examined is loaded into the program. It is first converted to a 16-bit picture to distinguish more grey levels. Next step is to apply the Gaussian blur to reduce the background noise. Once the background noise is subdued, auto threshold is performed to highlight the cells from the dark background. The particles are analysed, and a summary table is obtained. Table consists of columns named Major which corresponds to the cell length and Minor which corresponds to the width.

**
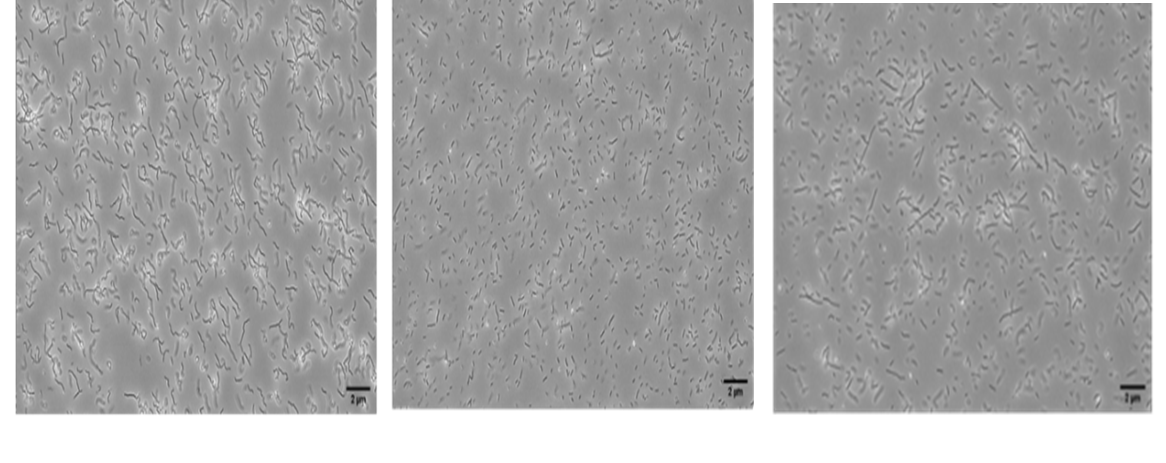
**

**Figure S3-1.** Microscopic pictures (40x magnification) for cells grown at 30℃, 37℃, and 44 ℃ (from left to right)

Cell volume was calculated using length and width of the cell, assuming cells to have a cylinder shape. It was calculated by using equation 1, where w is the width and l is the length of the cell that was obtained from ImageJ 1.52n analysis.

………………………. **Equation 1**

$$V=\frac{\pi w^{2}\left( \frac{l-w}{3} \right)}{4}$$

**Table S3-1.** Mean cell volume, and heterogeneity variables computed by microscopic analysis for conditions 1-15

| **Conditions** | **Mean cell volume (µm^3^)** | **Skewness** | **CV** | **rCV** |
| --- | --- | --- | --- | --- |
| 1 | 6.32 | 0.581 | 1.076 | 0.859 |
| 2 | 6.46 | 0.052 | 0.597 | 0.682 |
| 3 | 1.04 | 0.820 | 0.692 | 0.602 |
| 4 | 2.46 | 0.983 | 0.784 | 0.835 |
| 5 | 5.25 | 0.172 | 0.616 | 0.642 |
| 6 | 4.54 | 1.180 | 0.921 | 0.927 |
| 7 | 3.28 | 0.977 | 1.240 | 0.847 |
| 8 | 2.69 | 0.351 | 0.732 | 0.794 |
| 9 | 1.76 | 0.391 | 0.576 | 0.621 |
| 10 | 2.00 | 0.796 | 0.686 | 0.646 |
| 11 | 1.53 | 0.454 | 0.618 | 0.600 |
| 12 | 3.42 | 0.793 | 0.827 | 0.916 |
| 13 | 3.04 | 0.654 | 0.844 | 0.771 |
| 14 | 2.53 | 0.639 | 0.874 | 0.714 |
| 15 | 1.83 | 0.875 | 0.686 | 0.649 |

**Supplementary material S4. Flow cytometry analysis**

In this study, viability was studied using a mix of two dyes SYBR® Green I (Thermofisher Scientific, Sweden) and propidium iodide (PI). SYBR® Green I binds to DNA of all the cells and PI is extensively used to evaluate cell membrane integrity (Bunthof et al., 2001).

***Gating strategy to determine cell viability: FL1 vs FL3***

First, the sample is cleaned by gating based on the scattering profiles (FSC-H and SSC-H) to remove the noise. A log scale density plot of FL1-H vs FL3-H was obtained to visualize the fluorescence of the dyes. Intact and damaged cells are gated in such a way that most of the population in all the samples are captured (Nescerecka et al., 2016). Gating strategies and thresholds were kept the same for all the samples to attain comparable results.


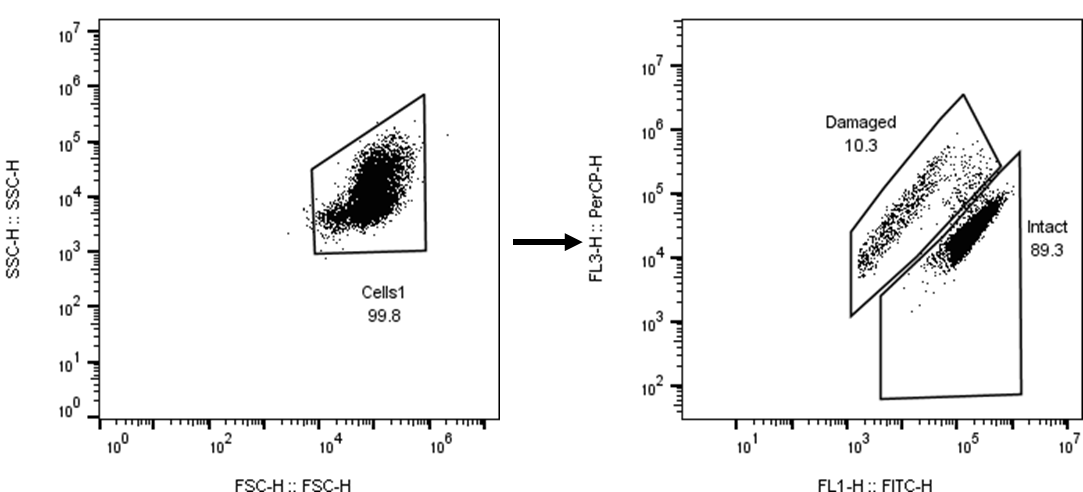


**Figure S4-1**. Graphical representation of gating strategy for denoising and subdivision into groups. Scattering profiles (FSC-H vs. SSC-H) were manual gated. A log scale density plot of FL1-H vs FL3-H was obtained to visualize the fluorescence of the dyes that was further sub grouped in to intact and damaged population.

***Gating strategy and calculations of morphological descriptors***

Mathematical functions such as Arithmetic mean, mode and median were used to evaluate average cell size based on the forward light scattering profile (Shapiro, 2000). Coefficient of variation (CV), Robust coefficient of variation (rCV) and skewness were evaluated for population heterogeneity. The FSC values required for the analysis were derived from the software FlowJo 10.6.

The skewness of the population distribution was performed by using mode and arithmetic mean. The mode of forward scatter was subtracted from the mean forward scatter resulting in positive value suggesting right skew or negative value suggesting negative skew (Heins et al., 2019).

**Table S4-1.** Morphological descriptors from FCM. Mean, skewness, CV, and rCV for FSC-H, and SSC-H, for conditions 1-15

| **Condition** | **Mean FSC-H** | **Mean SSC-H** | **Skewness**  **FSC-H** | **CV**  **FSC-H** | **rCV**  **FSC-H** | **Skewness**  **SSC-H** | **CV**  **SSC-H** | **rCV**  **SSC-H** |
| --- | --- | --- | --- | --- | --- | --- | --- | --- |
| 1 | 4.988 | 4.196 | 0.126 | 1.853 | 1.800 | 0.452 | 2.076 | 2.097 |
| 2 | 5.085 | 4.195 | 0.223 | 1.846 | 1.827 | 0.432 | 2.100 | 2.149 |
| 3 | 4.794 | 3.953 | -0.031 | 1.889 | 1.778 | 0.232 | 2.104 | 1.839 |
| 4 | 5.217 | 4.304 | 0.244 | 1.828 | 1.880 | 0.457 | 2.072 | 2.117 |
| 5 | 4.933 | 4.194 | 0.107 | 1.776 | 1.699 | 0.389 | 1.931 | 1.960 |
| 6 | 5.071 | 4.294 | 0.209 | 1.955 | 1.959 | 0.529 | 2.068 | 2.143 |
| 7 | 4.766 | 4.043 | 0.070 | 1.711 | 1.562 | 0.281 | 1.955 | 1.881 |
| 8 | 5.084 | 4.133 | 0.111 | 1.772 | 1.702 | 0.267 | 2.029 | 1.978 |
| 9 | 4.885 | 4.045 | 0.041 | 1.624 | 1.500 | 0.282 | 1.980 | 1.924 |
| 10 | 4.820 | 3.976 | -0.061 | 1.897 | 1.839 | 0.213 | 2.124 | 1.799 |
| 11 | 4.854 | 4.037 | 0.010 | 1.683 | 1.529 | 0.275 | 2.255 | 1.894 |
| 12 | 5.060 | 4.157 | 0.123 | 1.819 | 1.723 | 0.414 | 2.041 | 2.093 |
| 13 | 4.795 | 4.018 | -0.030 | 2.037 | 1.874 | 0.270 | 2.771 | 1.830 |
| 14 | 4.774 | 3.992 | -0.033 | 2.029 | 1.866 | 0.267 | 2.710 | 1.802 |
| 15 | 4.778 | 3.988 | -0.029 | 2.021 | 1.862 | 0.240 | 2.785 | 1.805 |

**Table S4-2.** Mean FSC, Mean SSC and Mean pulse width for cells cultivated at 30 ℃, 37 ℃ and 44 ℃.

| **Experiment** | **FSC-H mean** | **SSC-H mean** | **Mean pulse width** |
| --- | --- | --- | --- |
| 30 ℃ | 5.028 ± 0.003 | 4.321 ± 0.006 | 2.025 ± 0.008 |
| 37 ℃ | 4.755 ± 0.001 | 3.972 ± 0.000 | 1.939 ± 0.003 |
| 44 ℃ | 4.878 ± 0.032 | 4.072 ± 0.054 | 1.926 ± 0.006 |
| 30 ℃ FD | 5.026 ± 0.004 | 3.842 ± 0.009 | 1.994 ± 0.003 |
| 37℃ FD | 4.827 ± 0.001 | 4.008 ± 0.002 | 1.903 ± 0.00 |
| 44℃ FD | 4.815 ± 0.002 | 4.066 ± 0.002 | 1.863 ± 0.002 |


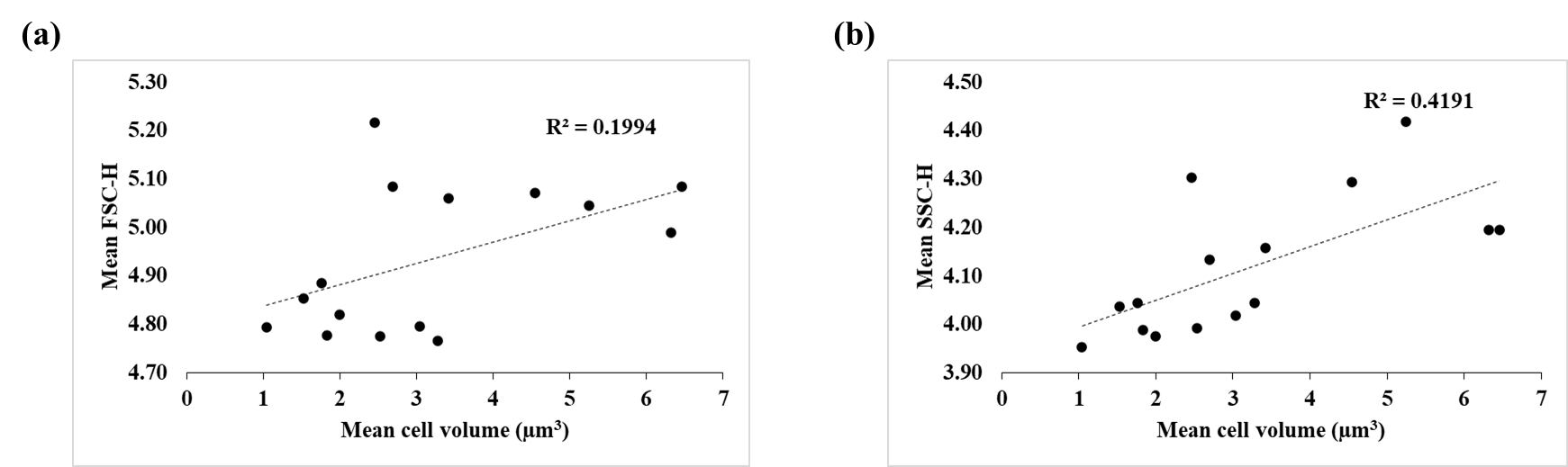


**Figure S4-2**. Correlations between Cell volume and (a) FSC-H mean, and (b) SSC-H mean. FSC-H mean obtained the poorest correlation with R^2^ value of 0.1994, and mean SSC-H mean with R^2^ value of 0.4191, respectively.


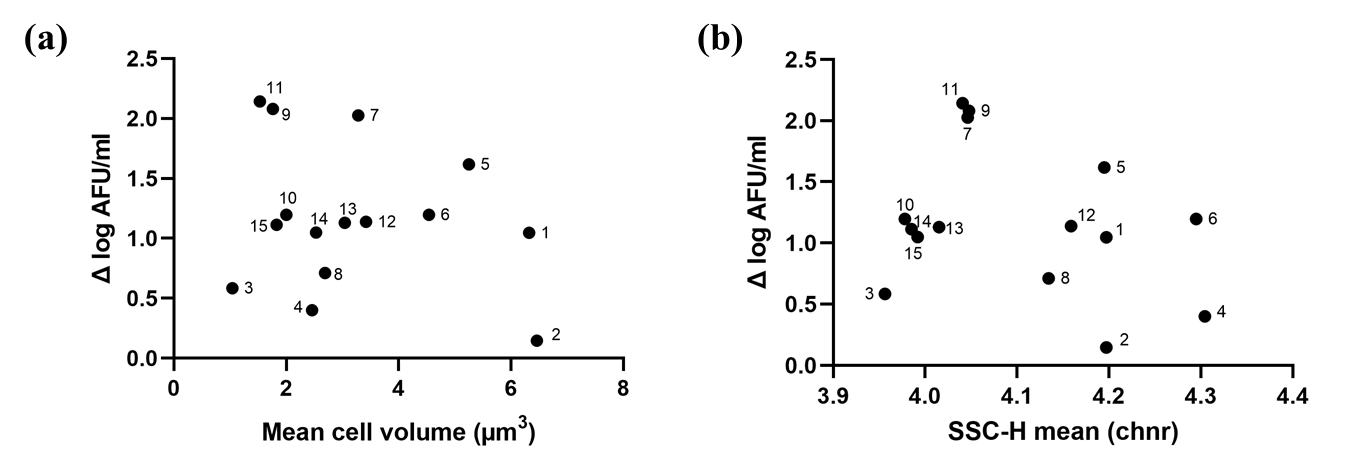


**Figure S4-3**. Morphology descriptors calculated for 15 conditions (a) Mean cell volume obtained from microscopic analysis, (b) SSC-H mean vs. growth (Δ log AFU/ml). Chnr-channel number.

**Table S4-3.** Correlation between growth (Δ log AFU/ml) and population heterogeneity descriptors. (ns= not significant, ** = very significant)

|  | Skewness (FSC-H) | rCV (FSC-H) | CV (FSC-H) |
| --- | --- | --- | --- |
| Pearson correlation coefficient r | -0.3601 | -0.7137 | -0.4968 |
| p- value | 0.1874 | 0.0028 | 0.0596 |
| p- value summary | ns | ** | ns |
| Significant? (alpha = 0.05) | No | Yes | No |

***Gating strategy for subpopulations based on pulse-width***

Heterogeneity was also assessed using pulse width descriptor. Peaks were gated using the create gates on peaks function. Five gates were found, and the gating was kept the same for all the samples to attain comparable results.


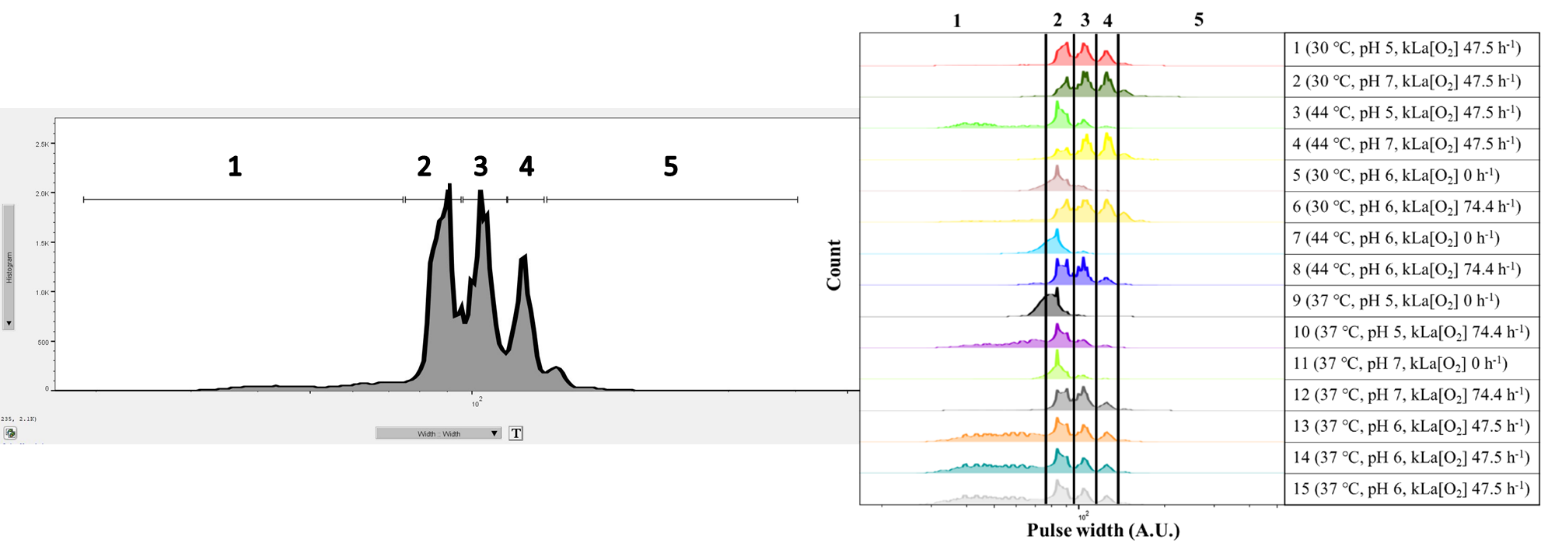


**Figure S4-4.** Gating performed based on peaks using FlowJo software on pulse width parameter. Histogram displaying gating performed for 15 conditions for total cells. Black line separates gates from each other.

Populations were divided based on pulse width, and tabulated. Also, individual populations such as intact and damaged cells were subjected to the same gating strategy.

**Table S4-4.** Population distribution based on pulse width gating for conditions 1-15

| **Condition** | **Total cells (%)** | | | | | **Damaged cells (%)** | | | | | **Intact cells (%)** | | | | | |
| --- | --- | --- | --- | --- | --- | --- | --- | --- | --- | --- | --- | --- | --- | --- | --- | --- |
|  | **Gate 1** | **Gate 2** | **Gate 3** | **Gate 4** | **Gate 5** | **Gate 1** | **Gate 2** | **Gate 3** | **Gate 4** | **Gate 5** | **Gate 1** | **Gate 2** | **Gate 3** | **Gate 4** | **Gate 5** |  |
| 1 | 7.55 | 36.2 | 31.2 | 18.4 | 4.54 | 65.5 | 18.6 | 8.93 | 3.75 | 1.62 | 0.32 | 38.4 | 34 | 20.2 | 4.86 |  |
| 2 | 2.7 | 23.4 | 32.1 | 26.7 | 12.7 | 38.1 | 33.1 | 13 | 6.45 | 5.42 | 1.37 | 23.1 | 32.8 | 27.5 | 12.9 |  |
| 3 | 42.1 | 40.6 | 11.9 | 3.35 | 0.9 | 88.3 | 7.78 | 2.16 | 0.76 | 0.29 | 1.87 | 69.2 | 20.4 | 5.59 | 1.43 |  |
| 4 | 3.13 | 22.8 | 31.9 | 29.8 | 10.2 | 35.4 | 31.3 | 18.4 | 8.93 | 3.06 | 0.81 | 22.2 | 32.9 | 31.4 | 10.7 |  |
| 5 | 14.2 | 66.3 | 13.5 | 1.83 | 0.43 | 44.1 | 42.4 | 8.62 | 1.44 | 0.57 | 9.8 | 69.8 | 14.2 | 1.88 | 0.41 |  |
| 6 | 14.6 | 24.7 | 26.7 | 20.7 | 10.6 | 61.1 | 20.4 | 9.23 | 4.46 | 2.65 | 0.63 | 26 | 32 | 25.7 | 12.9 |  |
| 7 | 21.2 | 67.3 | 6.37 | 0.54 | 0.39 | 54.9 | 35.7 | 5.1 | 0.8 | 0.52 | 13 | 75 | 6.65 | 0.47 | 0.36 |  |
| 8 | 5.77 | 44.8 | 32.8 | 10.9 | 3.25 | 29.1 | 38 | 19.8 | 7.02 | 3 | 0.3 | 46.5 | 35.9 | 11.8 | 3.28 |  |
| 9 | 24.6 | 65.7 | 2.68 | 0.6 | 0.65 | 59.4 | 30.8 | 3.72 | 1.21 | 1.04 | 22.7 | 67.7 | 2.62 | 0.56 | 0.62 |  |
| 10 | 44.1 | 36.8 | 12.5 | 3.61 | 0.8 | 78.2 | 16.3 | 2.33 | 0.67 | 0.53 | 1.72 | 62.2 | 25.1 | 7.27 | 1.14 |  |
| 11 | 8.58 | 73.8 | 11.8 | 2.28 | 1.19 | 76.1 | 17.1 | 2.47 | 1.09 | 1.1 | 3.96 | 77.7 | 12.4 | 2.36 | 1.2 |  |
| 12 | 5.94 | 42 | 34.1 | 11.6 | 3.97 | 67.2 | 21.4 | 5.38 | 1.9 | 1.57 | 0.53 | 43.9 | 36.6 | 12.4 | 4.12 |  |
| 13 | 48 | 26.6 | 13.9 | 7.59 | 2.3 | 86.1 | 10 | 1.91 | 0.61 | 0.31 | 3.23 | 45.8 | 28.1 | 15.9 | 4.65 |  |
| 14 | 50.8 | 26.5 | 12.9 | 6.79 | 1.55 | 90 | 7.35 | 1.3 | 0.36 | 0.17 | 3.75 | 49.3 | 27 | 14.6 | 3.21 |  |
| 15 | 50 | 26.4 | 13.3 | 6.92 | 1.86 | 89.9 | 7.18 | 1.46 | 0.39 | 0.21 | 3.53 | 48.5 | 27.3 | 14.6 | 3.76 |  |

**Table S4-5.** Population distribution based on pulse width gating for cells cultivated at 30 ℃, 37 ℃ and 44 ℃.

| **condition** | **Total cells (%)** | | | | | **Damaged cells (%)** | | | | | **Intact cells (%)** | | | | | |
| --- | --- | --- | --- | --- | --- | --- | --- | --- | --- | --- | --- | --- | --- | --- | --- | --- |
|  | **Gate 1** | **Gate 2** | **Gate 3** | **Gate 4** | **Gate 5** | **Gate 1** | **Gate 2** | **Gate 3** | **Gate 4** | **Gate 5** | **Gate 1** | **Gate**  **2** | **Gate 3** | **Gate 4** | **Gate 5** |  |
| 30 ℃ | 5.53 ±  0.02 | 28.35 ±  1.9 | 31.25 ±  1.06 | 24.8 ±  1.56 | 7.39 ±  1.38 | 21.1 ±  0.35 | 26.2 ±  1.77 | 24.05 ±  0.21 | 18.8 ±  0.99 | 7.24 ±  1.15 | 0.75 ±  0.05 | 29.1 ±  1.98 | 33.6 ±  1.20 | 26.6 ±  1.84 | 7.165 ±  1.39 |  |
| 37 ℃ | 6.195 ±  0.02 | 76.55 ±  1.6 | 12.35 ±  1.20 | 2.305 ±  0.57 | 0.695 ±  0.04 | 59.75 ±  3.75 | 30.5 ±  2.76 | 4.03 ±  0.23 | 1.415 ±  0.16 | 0.765 ±  0.33 | 2.53 ±  0.34 | 79.65 ±  1.48 | 12.9 ±  1.34 | 2.36 ±  0.62 | 0.685 ±  0.01 |  |
| 44 ℃ | 25.9 ±  1.13 | 51.6 ±  3.54 | 16.35 ±  2.62 | 2.7 ±  1.24 | 0.53 ±  0.20 | 33.55 ±  4.88 | 47.05 ±  0.64 | 13.35 ±  2.62 | 2.16 ±  0.98 | 0.51 ±  0.16 | 0.52 ±  0.28 | 65.5 ±  10.89 | 27.1 ±  7.57 | 4.735 ±  3.01 | 0.55 ±  0.28 |  |
| 30 ℃ FD | 11.35 ±  0.64 | 35.9 ±  1.2 | 30.2 ±  0.71 | 14.65 ±  0.92 | 4.72 ±  0.25 | 17.7 ±  0.14 | 38 ±  1.13 | 24.7 ±  0.14 | 11.5 ±  0.71 | 4.915 ±  0.32 | 0.455 ±  0.02 | 32.55 ±  0.92 | 39.8 ±  0.14 | 19.9 ±  0.49 | 4.1 ±  0.16 |  |
| 37 ℃ FD | 36.5 ±  0.28 | 50.45 ±  0.2 | 4.66 ±  0.03 | 2.465 ±  0.01 | 2.92 ±  0.03 | 66.15 ±  0.35 | 20.55 ±  0.35 | 4.04 ±  0.06 | 3.425 ±  0.04 | 3.535 ±  0.02 | 9.75 ±  0.03 | 77.45 ±  0.07 | 5.21 ±  0.01 | 1.595 ±  0.02 | 2.36 ±  0.03 |  |
| 44 ℃ FD | 65.75 ±  1.20 | 25.7 ±  1.1 | 3.375 ±  0.04 | 1.13 ±  0.06 | 0.72 ±  0.06 | 67.15 ±  1.20 | 24.55 ±  1.20 | 3.02 ±  0.03 | 1.135 ±  0.06 | 0.735 ±  0.05 | 0.425 ±  0.08 | 76.75 ±  0.35 | 19.9 ±  0.57 | 0.91 ±  0.06 | 0.1425 ±  0.10 |  |

**
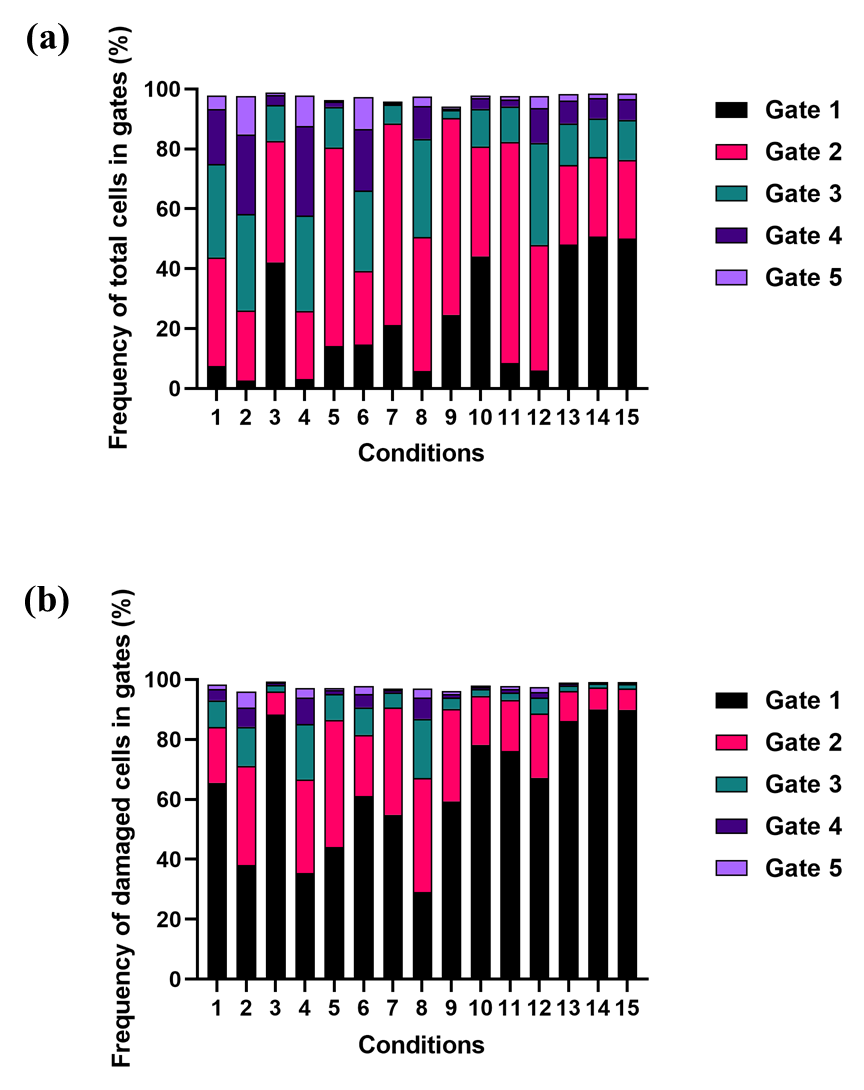
**

**Figure S4-5.** Population distribution based on pulse width peaks for conditions 1-15. (a) Total cells, (b) Damaged cells.


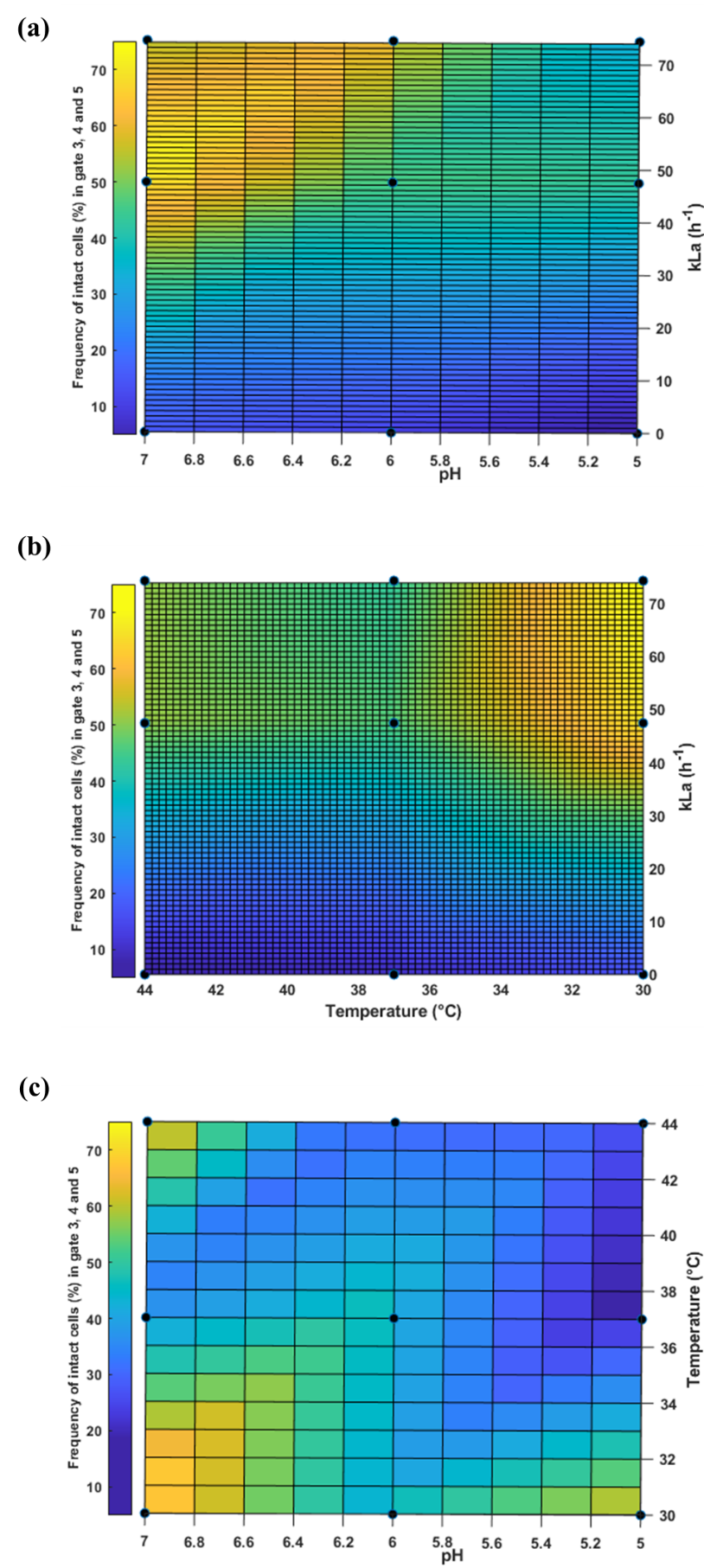


**Figure S4-6.** Correlation of frequency of intact cells (%) in gate 3, 4 and 5 with process variables. (**a**) pH and kLa, (**b**) temperature and kLa, (**c**) pH and temperature.

**Table S4-6.** Determination of significance for the change in frequency of pulse width distribution for samples before and after freeze drying. It was performed using unpaired t-test for samples before and after FD for a particular condition and specific gate. The yielding p-values are given below. It is performed for total cells and intact cells.

| **Total cells** | | | | | |
| --- | --- | --- | --- | --- | --- |
|  | **Gate 1** | **Gate 2** | **Gate 3** | **Gate 4** | **Gate 5** |
| **30 °C v 30 °C FD** | 0.0059 | 0.0432 | 0.3642 | 0.0155 | 0.1143 |
| **37 °C v 37 °C FD** | < 0.0001 | 0.002 | 0.012 | 0.731 | 0.0002 |
| **44 °C v 44 °C FD** | 0.0009 | 0.0101 | 0.0197 | 0.2167 | 0.3219 |

| **Intact cells** | | | | | |
| --- | --- | --- | --- | --- | --- |
|  | **Gate 1** | **Gate 2** | **Gate 3** | **Gate 4** | **Gate 5** |
| **30 °C v 30 °C FD** | 0.0157 | 0.1549 | 0.0188 | 0.0386 | 0.0906 |
| **37 °C v 37 °C FD** | 0.0011 | 0.1714 | 0.0147 | 0.2244 | 0.0002 |
| **44 °C v 44 °C FD** | 0.6919 | 0.2817 | 0.3091 | 0.2137 | 0.1933 |

**Supplementary material S5. Evaluation of correlations between freeze-thaw survivability and cell morphology descriptors**


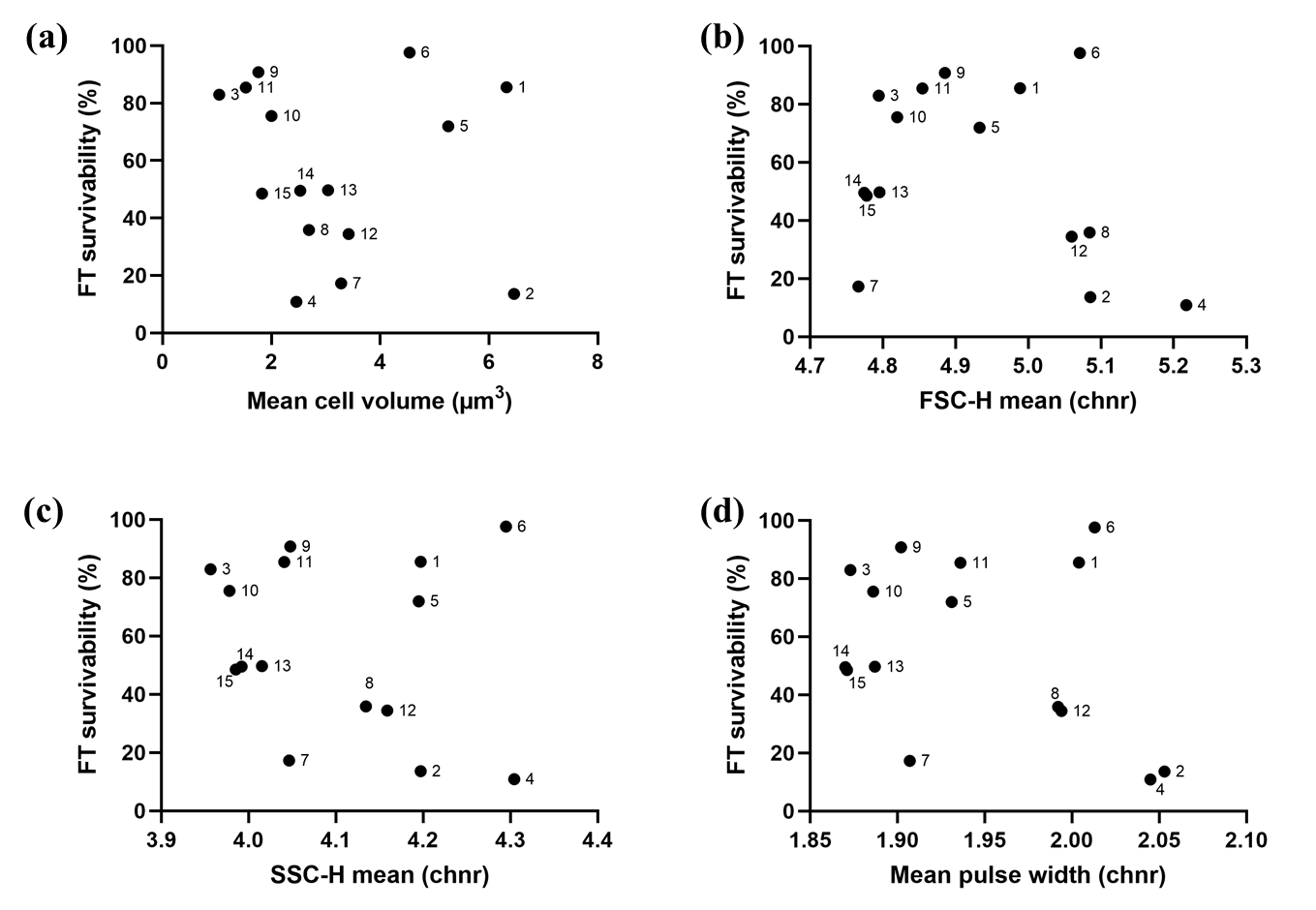


**
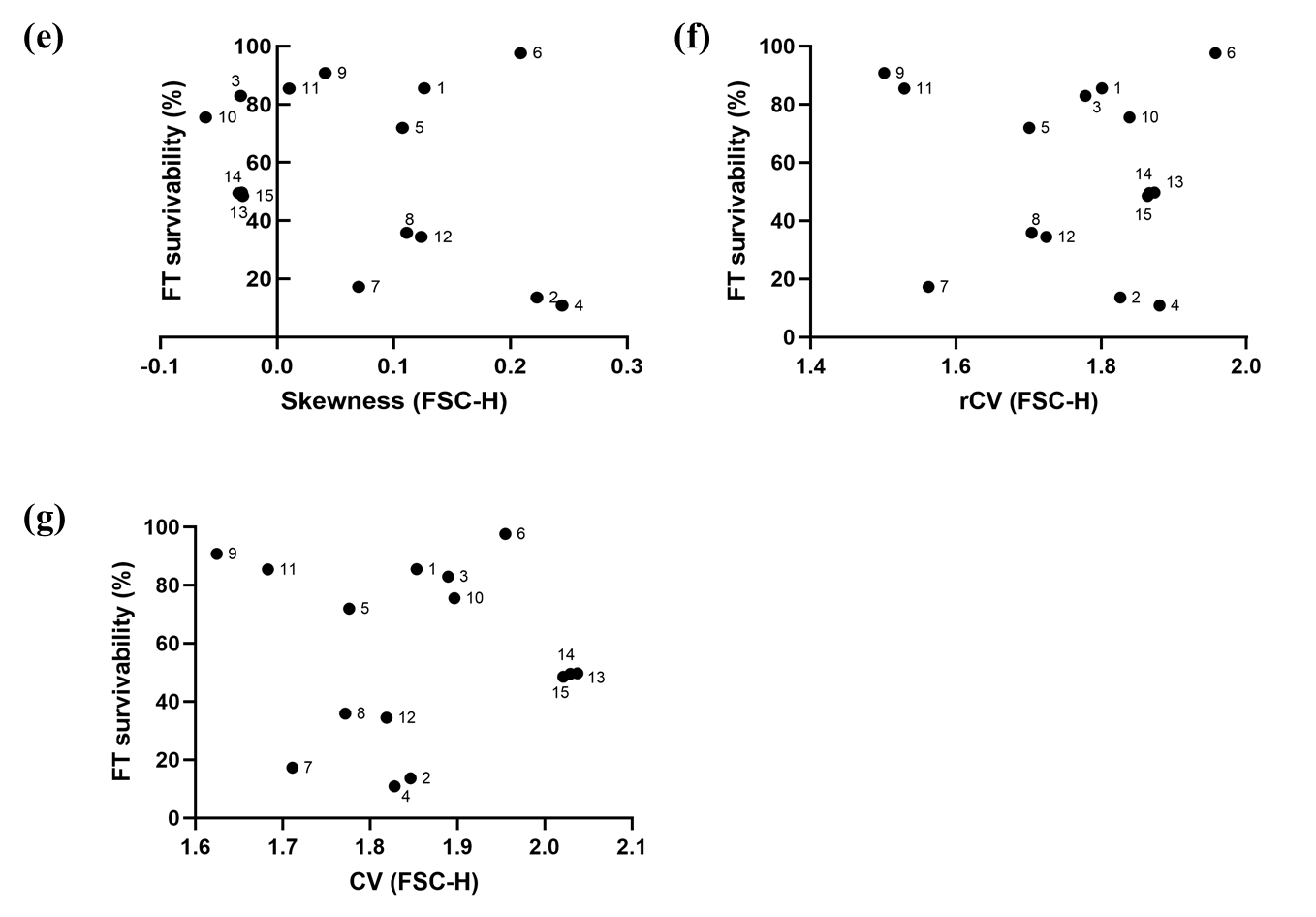
**

**Figure S5-1.** Morphology descriptors as a function of FT survivability for conditions 1-15 (a) Mean cell volume obtained from microscopic analysis, (b) Mean FSC, (c) Mean SSC, (d) Mean pulse width, (e) Skewness – FSC-H, (f) rCV – FSC-H, (g) CV – FSC-H.

**Table S5-1.** Correlation between freeze-thaw survivability and morphology descriptors. (ns= not significant)

|  | **FSC-H mean** | **SSC-H mean** | **Mean cell volume (µm^3^)** | **Mean pulse width (chnr)** | **Skewness** | **rCV** | **CV** |
| --- | --- | --- | --- | --- | --- | --- | --- |
| Pearson correlation coefficient r | -0.2954 | -0.1754 | -0.1231 | -0.317 | -0.3388 | -0.1073 | -0.0451 |
| p- value | 0.2852 | 0.5318 | 0.6621 | 0.2497 | 0.2168 | 0.7034 | 0.8731 |
